# O‐GlcNAcylation: cellular physiology and therapeutic target for human diseases

**DOI:** 10.1002/mco2.456

**Published:** 2023-12-19

**Authors:** Lin Ye, Wei Ding, Dandan Xiao, Yi Jia, Zhonghao Zhao, Xiang Ao, Jianxun Wang

**Affiliations:** ^1^ School of Basic Medicine Qingdao University Qingdao China; ^2^ The Affiliated Hospital of Qingdao University Qingdao Medical College Qingdao University Qingdao China

**Keywords:** disease, O‐GlcNAcylation, pathological processes, protein functionality, protein posttranslational modifications, therapeutic strategies

## Abstract

O‐linked‐β‐N‐acetylglucosamine (O‐GlcNAcylation) is a distinctive posttranslational protein modification involving the coordinated action of O‐GlcNAc transferase and O‐GlcNAcase, primarily targeting serine or threonine residues in various proteins. This modification impacts protein functionality, influencing stability, protein–protein interactions, and localization. Its interaction with other modifications such as phosphorylation and ubiquitination is becoming increasingly evident. Dysregulation of O‐GlcNAcylation is associated with numerous human diseases, including diabetes, nervous system degeneration, and cancers. This review extensively explores the regulatory mechanisms of O‐GlcNAcylation, its effects on cellular physiology, and its role in the pathogenesis of diseases. It examines the implications of aberrant O‐GlcNAcylation in diabetes and tumorigenesis, highlighting novel insights into its potential role in cardiovascular diseases. The review also discusses the interplay of O‐GlcNAcylation with other protein modifications and its impact on cell growth and metabolism. By synthesizing current research, this review elucidates the multifaceted roles of O‐GlcNAcylation, providing a comprehensive reference for future studies. It underscores the potential of targeting the O‐GlcNAcylation cycle in developing novel therapeutic strategies for various pathologies.

## Graphical abstract

The levels of O‐GlcNAcylation must be maintained within a certain “optimal zone” to preserve normal cellular function. Under stimuli or abnormal nutrient intake, the levels of O‐GlcNAcylation in cells might be disrupted due to the mutual regulation between OGT and OGA, leading to the loss of O‐GlcNAc homeostasis, which is a significant factor in the pathogenesis of various human diseases.

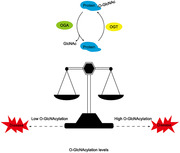

## 1 INTRODUCTION

Posttranslational modifications (PTMs) confer functional diversity to the proteome by modulating their molecular composition,^1^ which includes phosphorylcholination, glycosylation, ubiquitination, lipidation, methylation, N‐acetylation, and S‐palmitoylation, impact nearly every aspect of normal cell physiology and pathology. Hence, the identification and understanding of PTMs are crucial for studies in cellular biology, disease treatment, and prevention.^2^, ^3^, ^4^, ^5^ Therefore, recognizing and comprehending PTMs is vital in the research of cellular biology and the management and prevention of diseases.^6^

**FIGURE 1:**
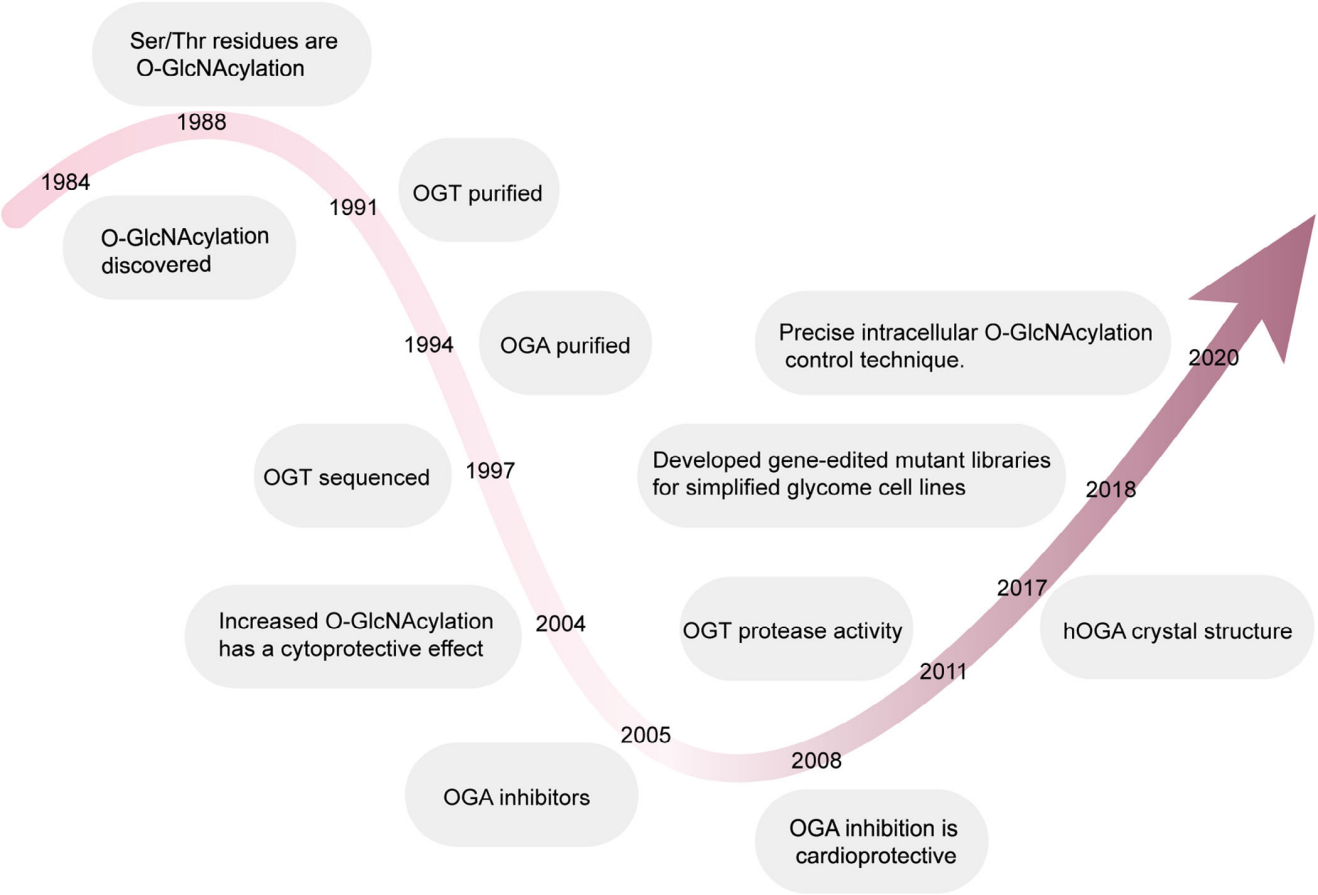
O‐GlcNAcylation annotation of key events in biology. So far, a detailed chronological overview of significant advances and discoveries in the field of O‐GlcNAcylation is not readily accessible. OGT, O‐GlcNAc transferase; OGA, O‐GlcNAcase; hOGA, human O‐GlcNAcase; Ser/Thr, serine/threonine.

Protein glycosylation, the modification of proteins via carbohydrates, is among the most prevalent PTMs across all cells and organisms.^7^, ^8^ In the early 20th century, carbohydrates were speculated to be key components of protein structures, yet the technology of the time could not provide conclusive evidence. It was not until the 1960s and 1970s that the intricate glycan structures and their functions on proteins were better understood. By the mid‐1980s, the dominant belief held that protein glycosylation was confined to extracellular proteins derived from the endoplasmic reticulum, Golgi apparatus, and secretory pathways. Yet, a study in 1984 by Torres and Hart altered this understanding.^9^ While aiming to describe N‐acetylglucosamine terminal residues on the surface of lymphocytes, they unexpectedly found that most of these terminal residues were intracellular, present as singly O‐linked GlcNAc monosaccharides. Holt and Hart further disclosed in 1986 that O‐GlcNAc‐modified proteins were distributed throughout almost all cellular compartments in rat liver cells, with particular enrichment in the cytoplasm and nucleus.^10^ The following year, a monoclonal antibody targeting the rat liver nuclear pore complex predominantly recognized O‐linked O‐GlcNAc moieties. Hanover and colleagues also identified nuclear pore proteins modified by O‐GlcNAc, although the functional consequences of this modification remained uncertain at the time.^11^ Concurrently, Holt and others identified serine and threonine (S/T) as the primary residues for O‐GlcNAc modifications.^12^ While O‐GlcNAc‐modified proteins were particularly abundant in the nucleus, such modifications were also found in anucleate red blood cells, indicating potential additional functions for this modification.^13^, ^14^, ^15^, ^16^ Subsequent research elucidated the key steps and factors involved in protein O‐GlcNAcylation. O‐GlcNAcylation attaches a singular GlcNAc molecule to the S/T residues of proteins through an O‐type‐β‐glycosidic linkage. The enzymes O‐GlcNAc transferase (OGT) and O‐GlcNAcase (OGA) are responsible for the incorporation and elimination of the sugar molecule, respectively. OGT mainly assists in the incorporation of β‐O‐GlcNAcylation, whereas OGA drives its hydrolytic removal (Figure  1).^17^, ^18^, ^19^

Since the initial identification of O‐GlcNAc‐modified proteins, they have been detected in all multicellular organisms, some bacteria, and viruses. In mammals, the O‐GlcNAc pathway is of paramount importance, as evidenced by the fact that removing the OGT gene results in embryonic fatality in mice.^20^ OGT and OGA have a widespread presence in mammalian organisms, and O‐GlcNAcylation occurs in proteins belonging to different functional categories. In light of the important function of O‐GlcNAcylation in cellular processes, its dysregulation is implicated in different pathophysiological contexts, including diabetes, cancer, cardiovascular diseases (CVDs), and neurodegenerative disorders. This review is intended to comprehensively investigate the connection between O‐GlcNAcylation and occurrences of human diseases, highlighting the key role of O‐GlcNAc‐modified proteins in disease contexts and discussing the current understanding of the O‐GlcNAcylation cycle. We will first outline the basic biology of O‐GlcNAcylation and its role in different cellular processes, followed by a detailed examination of its involvement in various diseases. Last, we will discuss the current challenges in research and future directions, aiming for a more comprehensive understanding of the role of O‐GlcNAcylation in disease onset and progression, and providing a scientific basis for developing new therapeutic strategies. This review offers readers a comprehensive framework to understand the complex role of O‐GlcNAcylation in disease development and the future research directions in this field, establishing a clear logical framework for readers to understand the interconnections between different parts of the manuscript and its overall significance.

## 2 REGULATION OF O‐GlcNAcylation

In this section, we focus on the key pathways and molecular mechanisms of O‐GlcNAcylation, with particular emphasis on the hexosamine biosynthetic pathway (HBP) and its central role in O‐GlcNAcylation. HBP not only provides the necessary substrates for O‐GlcNAcylation but is also the main pathway for the production of the crucial substrate UDP‐GlcNAc. We will delve into the initial steps of the HBP, including the uptake and transformation of glucose, as well as the synthesis mechanisms of UDP‐GlcNAc. Additionally, this section will thoroughly discuss the structural features, regulatory mechanisms, and their impacts on the protein functionality of OGT and OGA, highlighting the crucial role of OGT in catalyzing the addition of β‐N‐acetyl‐d‐glucosamine to proteins. OGA, a hexosaminidase first identified by Dong and Hart in 1994, will also be examined for its structural characteristics, including its gene encoding and functional domain composition in humans, and its necessity as a dimer in biological systems. This section will also cover how OGA works in conjunction with OGT to regulate transitions in O‐GlcNAcylation, which is essential for understanding the biological functions and potential therapeutic applications of OGT and OGA.

### 2.1 HBP

**FIGURE 2:**
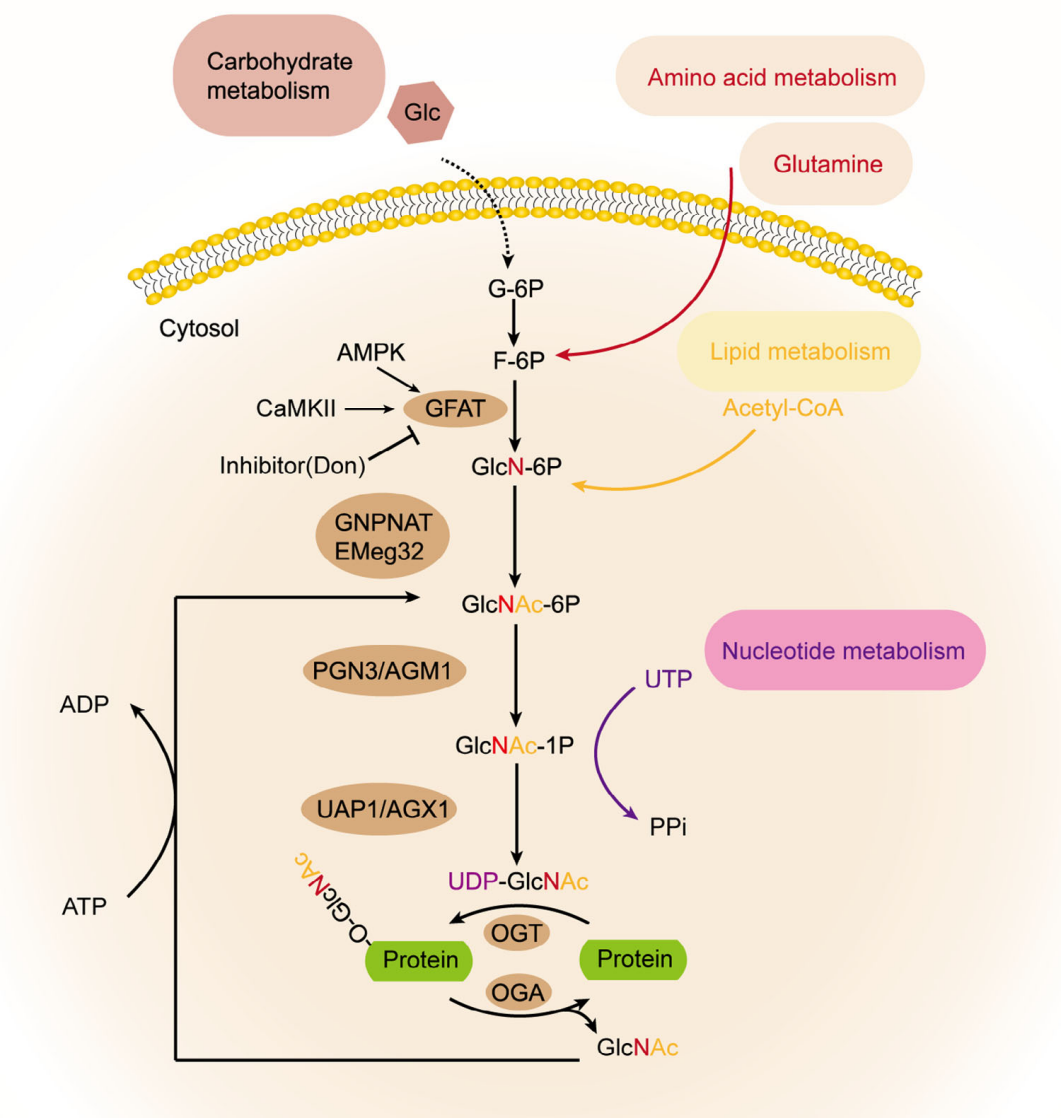
Overview of hexosamine biosynthesis pathway (HBP) and O‐GlcNAcylation. HBP integrates four metabolic pathways, including carbohydrates (glucose), amino acids (glutamine), lipids (acetyl‐CoA), and UTP. Glucose becomes F‐6P in the first two steps shared by HBP and glycolytic pathways. Only 2−3% of F‐6P enters HBP, where it binds to glutamine, acetyl‐CoA, and UTP to form UDP‐GlcNAc. OGT catalyzes the partial transfer of GlcNAc to Ser or Thr sites on protein substrates, while OGA can remove GlcNAc. Free GlcNAcs can be returned to HBP via a salvage pathway. ADP, adenosine diphosphate; AMPK, AMPK‐activated protein kinase; ATP, adenosine triphosphate; CaMKII, calcium/calmodulin (Ca^2+^/CaM)‐dependent protein kinase II; Emeg32, glucosamine 6‐phosphate N‐acetyltransferase; F‐6P, fructose‐6‐phosphate; GFAT, glucosamine fructose‐6‐phosphate amidotransferase; Glc, glucose; GlcNAc‐1P, N‐acetylglucosamine‐1‐phosphate; G‐6P, glucosamine‐6‐phosphate, GlcNAc‐6P, N‐acetylglucosamine‐6‐phosphate; GlcN‐6P, glucosamine‐6‐phosphate; PGN3/AGM1, phosphoglucomutase 3; PPi, pyrophosphate; UAP1/AGX1, UDP‐N‐acetylglucosamine pyrophosphorylase 1; UDP‐GlcNAc, uridine diphosphate N‐acetylgalactosamine; UTP, uridine triphosphate.

The HBP is intricately linked to O‐GlcNAcylation, wherein the HBP provides the essential substrate for O‐GlcNAcylation and is the primary route for producing the critical substrate, UDP‐GlcNAc.^21^, ^22^, ^23^ The HBP begins with the uptake of glucose, which is subsequently converted to fructose‐6‐phosphate through several reactions. Fructose‐6‐phosphate then reacts with glutamine to produce glucosamine‐6‐phosphate, a step catalyzed by the enzyme glutamine‐fructose‐6‐phosphate transaminase 1. Glucosamine‐6‐phosphate undergoes a sequence of enzymatic reactions leading to the formation of UDP‐GlcNAc. Specifically, glucosamine‐6‐phosphate is first N‐acetylated to form N‐acetylglucosamine‐6‐phosphate, which is then transformed into N‐glucosamine‐1‐phosphate, culminating in the formation of UDP‐GlcNAc. Once generated, UDP‐GlcNAc serves as a substrate for OGT, which adds a GlcNAc monomer to the S/T residues of proteins, resulting in O‐GlcNAcylation. In addition, OGA is responsible for removing GlcNAc from proteins, thereby facilitating the dynamic regulation of O‐GlcNAcylation (Figure 2).

In both mice and humans, two distinct subtypes of GFAT (GFAT1 and GFAT2) exist, located on different chromosomes and encoded by separate genes. Furthermore, each subtype displays a different tissue distribution.^24^ In the central nervous system, GFAT2 exhibits higher expression levels compared with GFAT1, whereas GFAT1 is produced at greater quantities than GFAT2 in various other bodily tissues.^25^ Studies indicate that in mammals, GFAT exists in a tetrameric form and its functioning is regulated by the presence of glucose. Additionally, glucosamine‐6‐phosphate and UDP‐GlcNAc have been identified as allosteric inhibitors of GFAT in mammals.^25^ GFAT is also subject to regulation via PTMs, and over 20 phosphorylation sites have been identified to date. GFAT1 and GFAT2 can be regulated by phosphorylation at different sites by cAMP‐dependent protein kinase A (PKA). Specifically, PKA phosphorylates GFAT1 at Ser‐205 and GFAT2 at Ser‐235 to modulate their activity.^26^, ^27^ In addition, the Ser‐243 site of GFAT1 can be phosphorylated by AMPK and Ca ^2+^/calmodulin‐dependent kinase (CaMKII; Figure 2).^28^, ^29^ Nonetheless, there are inconsistent findings regarding the impact of phosphorylation on GFAT's activity, potentially attributable to variations specific to different isotypes. Specifically, in the heart, it has been found that the activity of GFAT1 is significantly inhibited after phosphorylation by AMPK.^28^ It has been observed that transcription factors can also influence the activity of GFAT and PTMs. For instance, specific protein 1 (Sp1) and activation of transcription factor 4 (ATF4) have been shown to regulate the transcriptional activity of GFAT. However, the extent of their effect on GFAT activity in different cell types remains to be investigated.^30^, ^31^ There are also some recognized inhibitors of GFAT.^32^, ^33^ Take azeserine and 6‐diazo‐5‐oxo‐l‐norleucine (DON), for instance. However, a shared drawback among them is their lack of specificity and restricted applicability (Figure 2).

Glucosamine‐6‐phosphate N‐acetyltransferase (GNPNAT or GNA1), identified as Emeg32 in mice, is involved in the conversion of glucosamine‐6‐phosphate to N‐acetylglucosamine‐6‐phosphate through acetyl‐CoA. The resulting product is then isomerized to N‐acetylglucosamine‐l‐phosphate by phosphoglucosamine mutase (PGM). Subsequently, the enzyme UDP‐N‐acetylglucosamine pyrophosphorylase, also recognized as UDP‐N‐acetylglucosamine pyrophosphorylase (UAP1), facilitates the transformation of N‐acetylglucosamine‐1‐phosphate into UDP‐GlcNAc. Besides its role in a new synthesis, UDP‐GlcNAc can be replenished via two other compensatory pathways.^34^, ^35^ The first remedial pathway involves the phosphorylation of O‐GlcNAc by N‐acetylglucosamine kinase, forming N‐acetylglucosamine‐6‐phosphate. In the alternate pathway, N‐acetyl‐d‐galactosamine is converted to N‐acetylglucosamine‐1‐phosphate and UDP‐N‐acetylgalactosamine, and subsequently to UDP‐GlcNAc through a series of different enzymes. Researchers have speculated about the glucose fraction of HBP metabolism in fat cells. Direct measurement of glucose flow through The study of the HBP in comparison with other glucose metabolic routes was carried out using either radioisotope or stable isotope methods in cultured cells or whole organ systems. Using ^13^C6 glucose labeling in cultured new myocytes, Gibb and colleagues^36^ noted that the HBP metabolizes glucose more effectively than the pentose phosphate pathway, indicating that glucose processing through the HBP might be significantly greater than earlier assessments had suggested. However, this measurement was conducted under the low energy requirements of cell culture, and the energy requirements of new myocytes in reality are significantly different from those of adult hearts.^36^ Recently, Olson et al.^37^ developed an LC/MS technique. In isolated perfusion working hearts, the flow of HBP is measured to be 2.5 nmol/g/min, This represents merely 0.003–0.006% of the glycolytic flow, a figure that is substantially lower than prior estimates, possibly as a consequence of the heart's elevated metabolic rate. Furthermore, when the glucose concentration rises from 5 to 25 mM, it is alterations in glycolysis, not in the HBP flux rate, that lead to shifts in the relative flux of HBP compared with glycolysis. This highlights the limitations of HBP flux testing, which is evaluated as part of glycolysis. Despite the many unknowns regarding the regulation of HBP pathways, the development of new technologies has made it possible to directly measure HBP fluxes in different biological systems.

### 2.2 OGT: structural insights, regulatory mechanisms, and functional impacts

**FIGURE 3:**
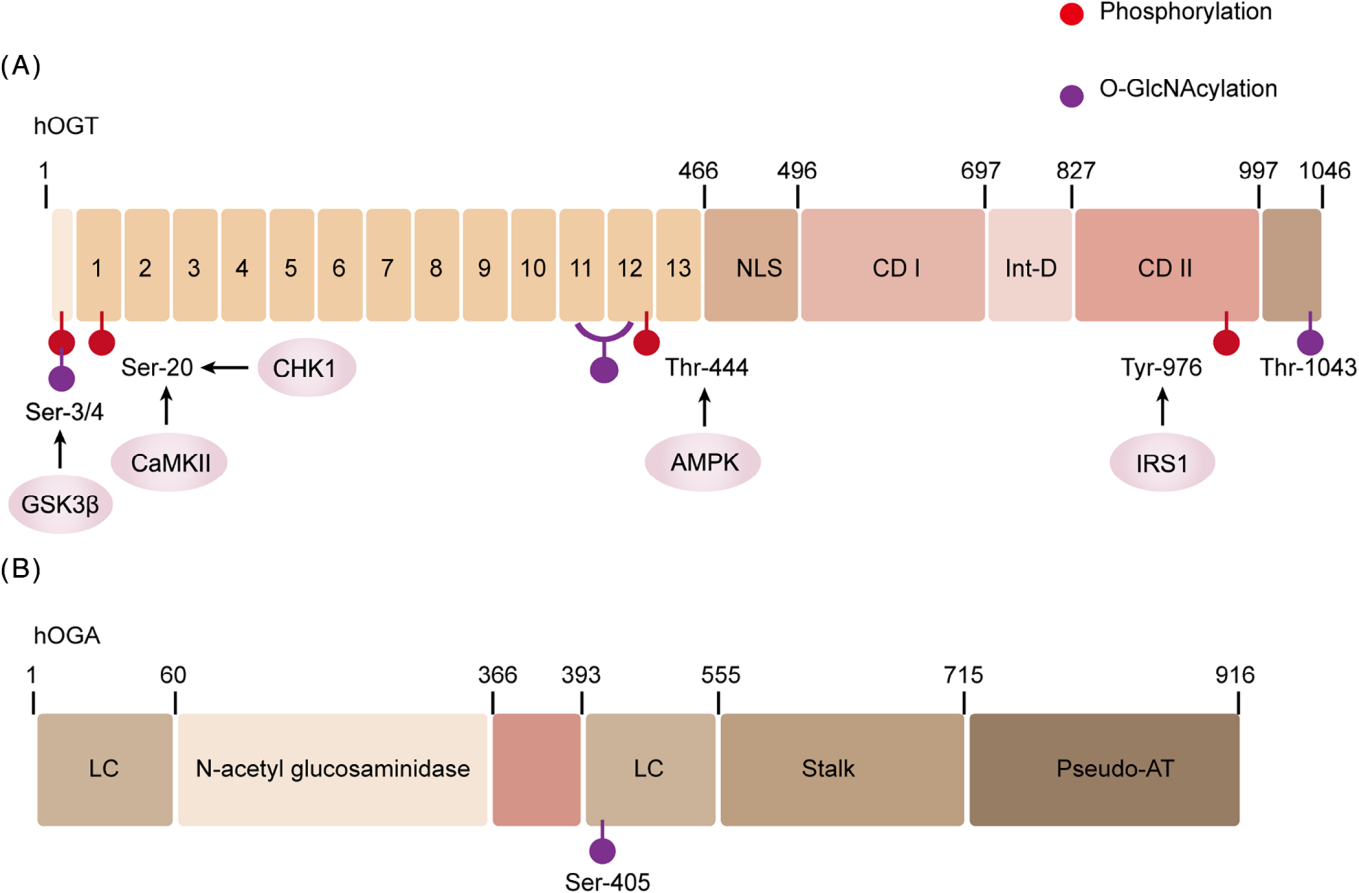
Configuration and posttranslational alterations of human OGT and OGA. The phosphorylation sites of the specified kinase are indicated by red circles, and all recognized phosphorylation sites are listed in the figure below. O‐GlcNAcylation sites are shown as purple squares. (A) OGT structure. The N‐terminal region of human OGT consists of TPRs (tetratricopeptide repeats) and an NLS (nuclear localization signal). The catalytic region comprises an N‐cat (N‐terminal catalytic domain) and an Int‐D (intervening domain. (B) OGA structure. It is composed of a catalytic domain in the N‐terminal region, two stalk domains (including N‐terminal and C‐terminal), and two LC (low complexity) regions. CaMKII, calcium/calmodulin (Ca^2+^/CaM)‐dependent protein kinase II; CD, catalytic domain; CHK1, checkpoint kinase‐1; GSK3β, glycogen synthase kinase 3β; Int‐D, intermediate domain; IRS1, insulin receptor substrate‐1; LC, low complexity area; NLS, nuclear localization sequence; TPR, tetrapeptide repeats.

OGT was initially extracted from the liver of rats in 1992 and reached by cloning 5 years later.^38^, ^39^, ^40^ The study revealed that the OGT gene is situated on the X chromosome and encodes a protein that comprises two primary domains. The initial domain includes a series of tetrapeptide repeats (TPRs), and the count of TPRs differs based on the organism.^40^ TPR is a spiral‐to‐helical motif generally involved in regulating protein interactions.^41^, ^42^ In humans, the full‐length OGT protein is present in both the nucleus and the cell and comprises 13 intact TPR repeats in its N‐terminal domain (Figure 3). Additionally, there are two other subtypes: one subtype contains a mitochondrial localization signal and 9 intact TPRs, while the other subtype, known as sOGT, has only 2.5 TPRs. The enzymatic region of OGT is located in the C‐terminus of the protein.^43^ OGT, an soluble enzyme, is present in both the cytoplasm and nucleus of metazoan cells.^44^, ^45^ OGT is the exclusive enzyme tasked with catalyzing a considerable array of PTMs by attaching β‐linked N‐acetyl‐d‐glucosamine to S/T residues of nucleoproteins or cytoplasmic proteins.^46^, ^47^, ^48^ However, in the case of mammals, OGT is present throughout the development of life.^49^, ^50^, ^51^ Research has demonstrated that nondividing cells can survive for extended periods without OGT, but their physiology is significantly disrupted, although apoptosis does not occur.^51^, ^52^, ^53^ The ablation of OGT in cardiomyocytes results in changes in cell size and increased heart failure (HF). As a result, mice with this genetic defect have a lower survival rate.^53^ However, it is interesting that cardiomyocytes show increased apoptosis markers after mitosis.^54^ Notably, the survival of the majority of mice with OGT ablation in cardiomyocytes suggests their ability to endure, albeit with the impaired performance of their typical physiological functions. It is important to recognize that phosphorylation of Ser, Thr, and Tyr residues can also regulate OGT activity and substrate recognition.^55^ Research has revealed that OGT can be phosphorylated at approximately 20 distinct sites, which can be identified through proteomic techniques. It has been observed that insulin can enhance OGT phosphorylation by activating the insulin receptor (IR), resulting in elevated OGT activity.^56^, ^57^ OGT is also a target of phosphorylation by glycogen synthase kinase (GSK)‐3β, which has been shown to enhance OGT activity.^58^ The phosphorylation of OGT at Thr‐444 by AMPK results in changes in its subcellular distribution and the targets it binds to for substrate interaction.^59^ Furthermore, OGT Ser‐20 is capable of phosphorylation by checkpoint kinase 1 (Chk1), which is necessary for cytoplasm division.^60^ Ser‐20 on OGT is another phosphorylation site targeted by CaMKII, and OGT's activity is enhanced following this phosphorylation.^61^ Apart from being phosphorylated, OGT itself can also be O‐GlcNAcylated. In particular, the O‐GlcNAcylation of Ser‐389, situated within the TPR domain, has been demonstrated to impact OGT's subcellular localization.^62^ O‐GlcNAcylation can also occur on serine 3/4 of OGT, although the specific biological implications of this modification remain to be studied (Figure 3). In addition to its intrinsic functions, OGT can also impact other proteins. Adaptor or scaffold proteins can interact with OGT and facilitate the recruitment of substrates for O‐GlcNAcylation. As an example, peroxisome proliferator‐activated receptor gamma coactivator 1‐α (PGC‐1α) is O‐GlcNAcylated by increased OGT activity during fasting in liver tissues. This modification leads to enhanced PGC‐1α stability and the upregulation of genes involved in gluconeogenesis.^63^ This also encompasses p38 MAPK, which is involved in directing OGT to the neurofilament heavy polypeptide,^64^ and REV‐ERBα inhibits the degradation of OGT.^65^ Interestingly, the OGT–OGA interaction may have increased the likelihood of the “O‐GlcNAcylationzyme” complex, consistent with that O‐GlcNAcylation is a rapidly reversible change.^66^ OGT knockout has been shown to downregulate O‐GlcNAcylation, resulting in increased interaction between α‐ketoglutaric acid, Hypoxia‐Inducible Factor‐1α (HIF‐1α) hydroxylation, and von Hippel‐Lindau protein, ultimately leading to HIF‐1α degradation, endoplasmic reticulum stress and apoptosis mediated by C/EBP homologous protein (CHOP) are also involved in this process.^67^ Furthermore, the research suggests that the PI3K–mTOR–MYC signaling pathway plays a critical role in the upregulation of OGT and the subsequent O‐GlcNAcylation process. Following treatment with PI3K and mTOR inhibitors, there is a decrease in OGT protein expression and O‐GlcNAcylation levels. Conversely, activation of AKT and mTOR can reverse this effect.^68^ The c‐MYC transcription factor, which is implicated in oncogenic properties and positioned downstream of the mTOR pathway, plays a role in governing the expression of the OGT protein.^68^, ^69^ Insulin exposure prompts the migration of OGT from the nucleus to the cytoplasm and plasma membrane, while variations in nutritional conditions bring about shifts in the allocation of OGT between the nucleus and cytoplasm.^56^, ^70^, ^71^ Besides functioning as a glycosyltransferase, OGT also displays protease activity. Transcriptional coregulator HCF1 as a proteolytic substrate.^72^, ^73^ Both UDP‐GlcNAc and OGT are required for HCF1 proteolysis, which is consistent with the concept that HCF1 can undergo O‐O‐GlcNAcylation.^74^ The Walker lab has identified several potential OGT inhibitors through high‐throughput screening.^75^ Two compounds TT04 and TT40, have irreversible effects.^76^ However, low solubility has also been shown.^77^ TT04 has been used in the laboratory as an OGT inhibitor.^78^, ^79^ Vocadlo and coworkers^80^ has developed an O‐GlcNAc substrate analog called 5‐thioglucosamine (5SGlcNAc). Due to its hydrophobic characteristics, acetylated 5S‐GlcNAc effortlessly penetrates cell membranes, undergoes transformation into UDP‐5S‐GlcNAc, and ultimately attaches to the catalytic site of OGT, resulting in the inhibition of its activity.

### 2.3 OGA: structural insights and regulatory mechanisms

OGA, first identified by Dong and Hart in 1994, is a hexosaminidase.^81^ After isolation from the rat spleen, it is also called NCOAT, nuclear plasma O‐GlcNAcylationase, and acetyltransferase.^82^, ^83^, ^84^ The gene for human OGA is situated on chromosome 10 and encodes a protein consisting of an NH2‐terminal glycosylhydrolase domain and a COOH‐terminal domain. Short amino acid motifs or single amino acids are repeated on both sides of the glycosylhydrolase domain. After the extensive region of low complexity is the stem domain, that interacts with the COOH‐terminal domain.^85^, ^86^ Studies on the OGA structure have revealed that the human OGA (hOGA) absence of the necessary residues to bind with acetyl‐CoA. Therefore, this region has been described as a “pseudo‐AT domain with no catalytic activity.”^87^ Selective splicing gives rise to short OGA (sOGA), which, however, does not possess a pseudo‐AT region and features a distinct amino acid COOH‐terminal region.^88^ In 2017, the crystal structure of hOGA was deciphered.^89^, ^90^, ^91^ Recent studies have demonstrated that hOGA forms an obligatory homodimer indicating that OGA functions biologically as a dimer. The structural examination of OGA implies its potential to selectively eliminate O‐GlcNAcylation from specific sites, suggesting a possible involvement in regulating transitions in O‐GlcNAcylation in collaboration with OGT.^92^ Like to OGT, phosphorylation and O‐GlcNAcylation serve as regulatory mechanisms for OGA as well. Mass spectrometry mapping of proteins has identified at least 20 different phosphorylation sites on OGA at Ser, Thr, and Tyr residues; however, the impact of these modifications on OGA remains unclear. Interestingly, the OGA Ser‐405 site, that functions as the site where OGA interacts with OGT, is a site for O‐GlcNAcylation (Figure 3).^86^

## 3 FUNCTIONS OF O‐GlcNAcylation

### 3.1 Transcriptional regulation

**FIGURE 4:**
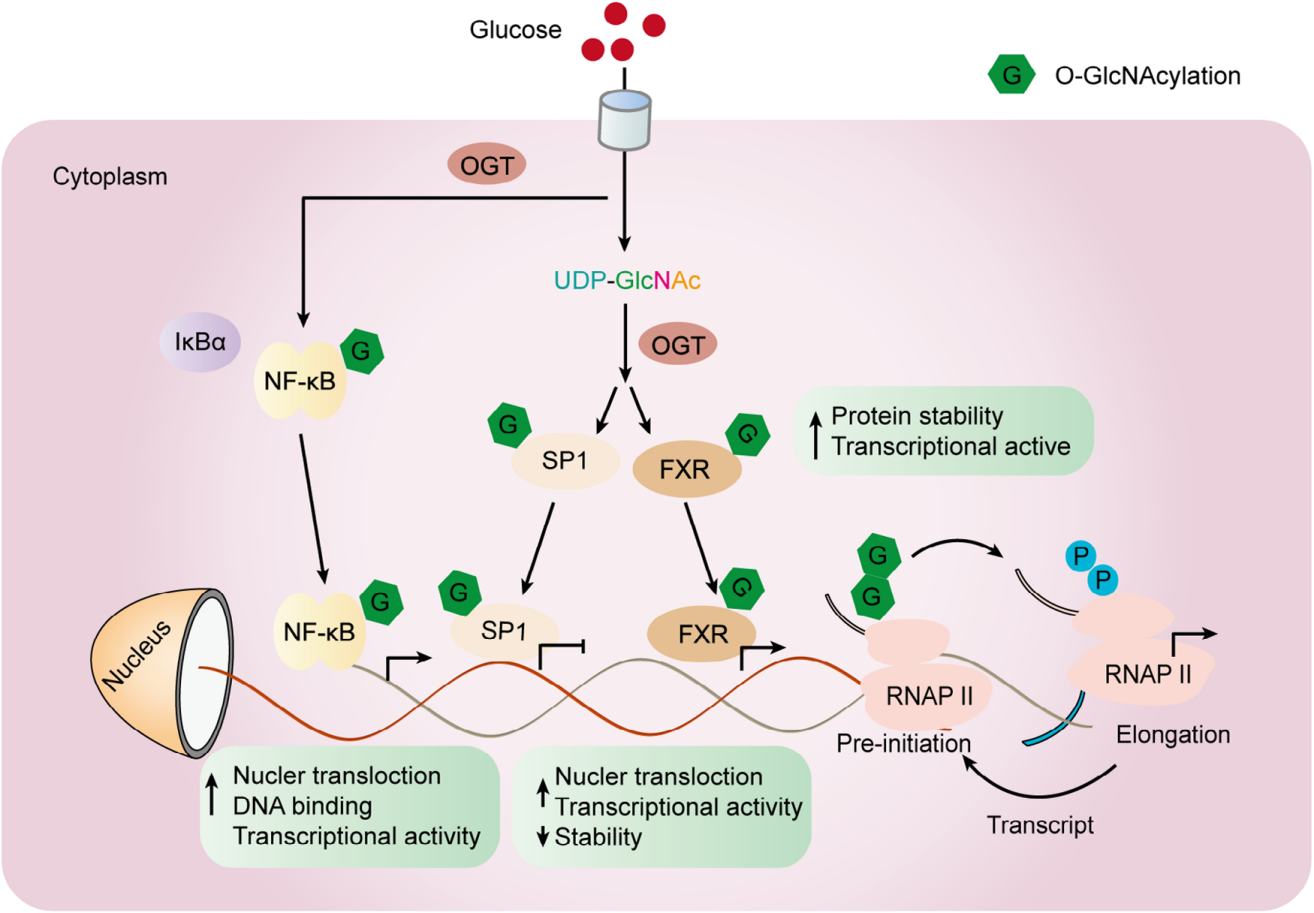
Transcription factor O‐GlcNAcylation and RNA polymerase II (Pol II) regulates transcriptional activation and inhibition. O‐GlcNAcylation of nuclear factor κB (NF‐κB) facilitates its movement to the nucleus (by inhibiting its interaction with IκBα) and amplifies its DNA binding as well as transcriptional functions. O‐GlcNAcylation of Sp1 increases its nuclear localization and stability, but at the same time inhibits its transactivation. FXR interacts with and is transformed by its O‐GlcNAcylation in its N‐terminal AF1 domain. Increased acylation of FXR O‐GlcNAcylation enhances FXR gene expression and protein stability in a cell type‐specific manner. The O‐GlcNAcylation of Pol II on its carboxyl‐terminal domain (CTD) is important for assembling preinitiation complexes at transcriptional initiation sites, while removal of O‐GlcNAcylation from Pol II CTD allows it to be dynamically phosphorylated during transcriptional initiation and extension. Therefore, mutual O‐GlcNAcylation and phosphorylation of Pol II CTD are critical for maintaining an undisturbed transcription cycle. GLC, glucose; UDP‐GlcNAc, uridine diphosphate N‐acetylglucosamine; IκBα, NF‐κB inhibitors α; Sp1, specific protein 1, FXR, farnesate X receptor; RNAPII, RNA polymerase II.

While both OGT and OGA can exist in both the nucleus and cytoplasm, studies have shown that OGT is predominantly located in the nucleus. At the same time, OGA is mainly localized in the cytoplasm.^93^ As the gene pool of O‐GlcNAcylation transcription factors and cofactors continues to expand, recognition of their biochemical processes is growing. As a demonstration, within immune cells, O‐GlcNAcylation of OGT catalyzes the activation of key transcription factors of T cells, namely nuclear factor 1 (NFA‐Tc1) and nuclear factor κB (NF‐κB), which plays a crucial role in initiating the activity of white blood cells. In liver cells, O‐GlcNAcylation of CRTC2, FOXO1, and PGC‐1α can modulate the expression of gluconeogenic genes (Table 1).^63^, ^94^, ^95^ Sp1 is a zinc‐finger transcription factor that can bind to the GC‐rich motifs of various promoters. A study conducted by Jackson revealed that an increase in O‐GlcNAcylation on human cells' Sp1 resulted in elevated transcriptional activity.^96^ The O‐GlcNAcylation modification protects Sp1 from proteasome degradation.^97^, ^98^ In addition, the O‐GlcNAcylation of Sp1 could disrupt interactions among transcription factors, suggesting that the process of O‐GlcNAcylation in transcription factors contributes to the intricate mechanisms governing gene expression.^99^, ^100^, ^101^ Regarding NF‐κB, O‐GlcNAcylation diminishes its binding to IκBα, this leads to an increased nuclear translocation and enhanced gene expression activity. On the other hand, the O‐GlcNAcylation of c‐Rel is essential for its ability to bind to DNA and carry out transcription functions.^102^ RNA polymerase II (Pol II) is recognized to undergo modification through O‐GlcNAcylation.^103^ The C‐terminal domain (CTD) of Pol II undergoes concurrent O‐GlcNAcylation and phosphorylation at Ser‐2/5 (Figure 4).

**TABLE 1:** List of selected transcription factors and transcriptional regulators undergoing O‐GlcNAcylation in diseases (1995–2023).

Protein	Modification sites	Protein function	References
C/EBβ	Ser‐180, Ser‐181	The reciprocal opposition between O‐GlcNAcylation and phosphorylation of C/EBPβ induces changes in its DNA binding and transactivation functions, thereby affecting the differentiation of adipocytes.	^104^, ^105^, ^106^
cMyc	Thr‐58	Decreases Thr‐58 phosphorylation and changes the ubiquitination level.	^107^, ^108^, ^109^, ^110^, ^111^, ^112^
CREB	Ser‐40	Represses CREB activity via a CRTC‐dependent mechanism.	^113^, ^114^, ^115^
ERα	Ser‐10, Thr‐50,Thr‐575	O‐GlcNAcylation may regulate mER‐α turnover.	^116^
ERRγ	Ser‐317, Ser‐319	ERRγ ubiquitination is reduced, leading to the stabilization of the receptor and an increased ability to induce gluconeogenesis.	^117^
KEAP1	Ser‐104	Ubiquitination and degradation of NRF2.	^118^
Oct4	Thr‐116, Thr‐225, Ser‐236, Ser‐288/889/890, Ser‐335, Ser‐349, Thr‐351, Thr‐352, Ser‐355, Ser‐359	Transcriptional activation of Oct4.	^119^, ^120^
P53	Ser‐139	O‐GlcNAcylation fortifies p53 by impeding its degradation through a ubiquitin‐dependent process.	^121^, ^122^, ^123^, ^124^
PGC‐1α	Ser‐333	Protect PGC‐1α from degradation and promote gluconeogenesis.	^125^
PPAR‐γ	Thr‐54	Transcriptional activity is reduced .	^126^
SIRT1	Ser‐549	enhance deacetylase activity.	^127^, ^128^, ^129^

The proteins listed in this table were primarily chosen based on their incorporation within the main content of the article. C/EBP, CCAAT/enhancer‐binding protein; cMyc, cellular‐myelocytomatosis viral oncogene; CREB, cyclic‐AMP response binding protein; ERα, estrogen receptor alpha; ERRγ, estrogen‐related receptor gamma; Keap1, Kelch‐1ike ECH‐associated protein l; Oct4, octamer‐binding transcription factor; p53, tumor protein P53; PPAR‐γ, peroxisome proliferator‐activated receptor gamma.

### 3.2 Calcium signaling

The interplay between Ca2+ signaling and protein O‐GlcNAcylation has gained considerable attention. In the cardiac system, O‐GlcNAcylation of CaMKII at Ser‐279 results in CaMKII initiation and heightened arrhythmias in diabetic patients.^130^ In the liver, CaMKII phosphorylates OGT, leads to elevated levels of O‐GlcNAcylation, thereby activating autophagy. PLB is also subject to O‐GlcNAcylation, with Ser‐16 being the most probable site, which diminishes PKA‐mediated PLB Ser‐16 phosphorylation by inhibiting OGA or increasing OGA PLB O‐GlcNAcylation levels under hyperglycemic conditions. Increased O‐GlcNAcylation levels correlate with reduced SERCA activity and enhanced SERCA‐PLB binding.^131^ The rise in PLB O‐GlcNAcylation might contribute to the delayed reuptake of Ca2+ by cardiac ER/SR in diabetic patients. Additionally, it is becoming more apparent that O‐GlcNAcylation levels are controlled in a manner dependent on Ca2+, like through the CaMKII/IV‐mediated phosphorylation of OGT, which leads to increased activity and higher levels of O‐GlcNAcylation.^132^

### 3.3 Cell survival/ferroptosis/autophagy

**FIGURE 5:**
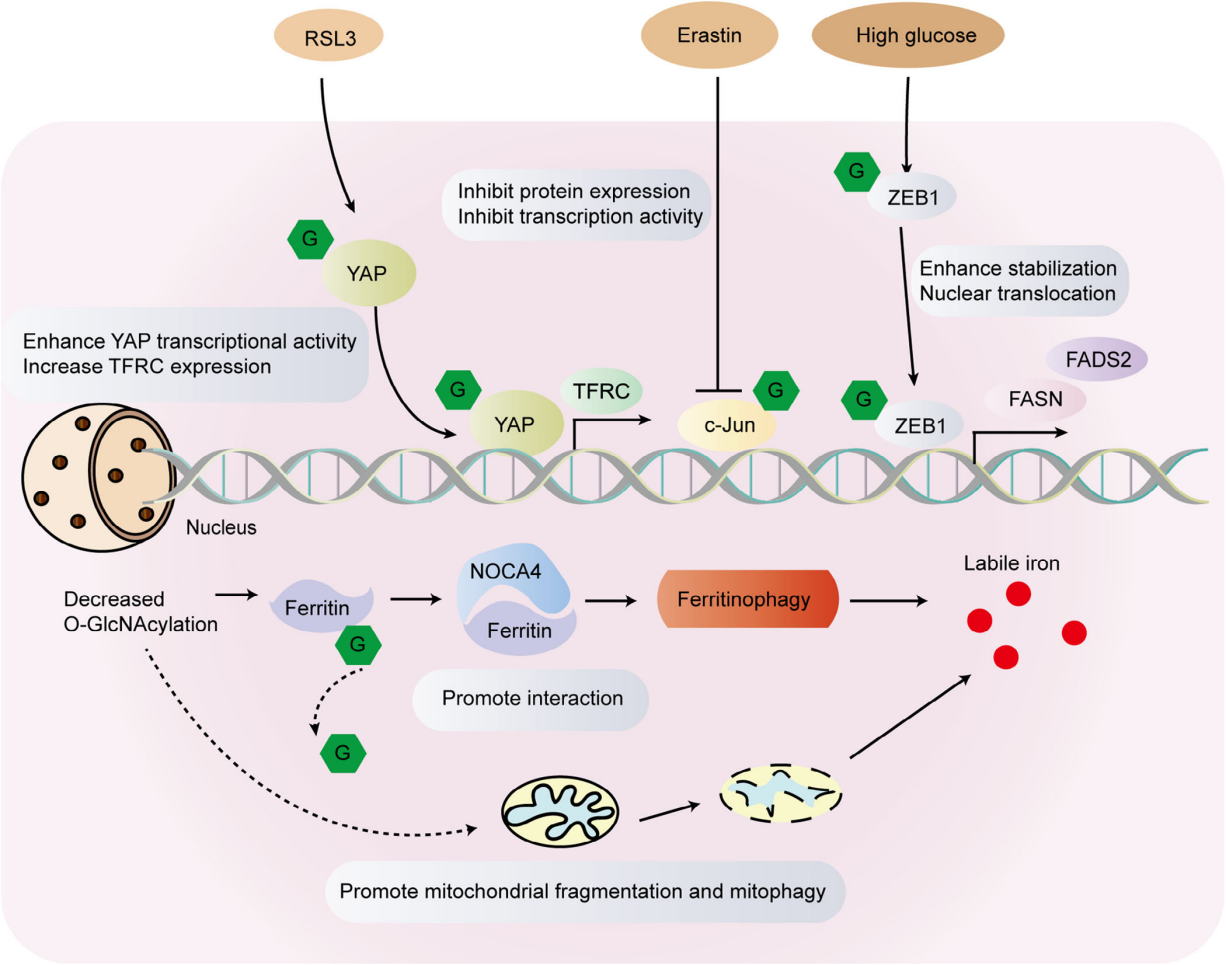
O‐GlcNAcylation in the ferroptosis signaling pathway. After YAP O‐GlcNAcylation, the transcription of TFRC was increased, and the iron concentration in the guide cells was increased. Erastin inhibits c‐jun's protein expression, transcriptional activity, and nuclear accumulation by inhibiting c‐jun O‐GlcNAcylation. After O‐GlcNAcylation, ZEB1 enhanced its stability and nuclear translocation, enhanced the transcriptional activity of adipoietic‐related genes, and finally led to ferroptosis. The de‐O‐GlcNAcylation of ferritin promotes their interaction with the ferritin phage receptor NCOA_4_, thereby accumulating iron. FADS2, fatty acid desaturase 2; FASN, fatty acid synthase; NCOA_4_, nuclear receptor coactivator 4; RSL3, ((1S,3R)‐RSL3) is an inhibitor of glutathione peroxidase 4 (GPX4); TFRC, transferrin receptor; YAP, Yes associated transcriptional regulator; ZEB1, zinc finger E‐Box binding homeobox 1.

Enhanced levels of O‐GlcNAcylation in tumor cells have been linked to cell death in pharmacological investigations of OGT or OGA inhibition. As such, it is increasingly crucial to comprehend the correlation between cell death and glycosylation. Siderozosis is a regulatory iron‐dependent cell death marked by the accumulation of lipid peroxidation. Experimental analyses have established that protein O‐GlcNAcylation, the primary glucose flux nutrient sensor, coordinates ferritin phage and mitochondrial autophagic ferroptosis.^133^, ^134^ Inhibition of O‐GlcNAcylation results in mitochondrial fragmentation and enhanced mitophagy, generating an additional pool of labile iron and rendering cells more susceptible to ferroptosis. De‐O‐GlcNAcylation of the ferritin heavy chain at Ser‐179 promotes its interaction with the ferritin phage receptor NCOA4, leading to iron accumulation (Figure 5).^135^ Under high glucose conditions, the O‐GlcNAcylation level in mesenchymal pancreatic cancer cells was linked to ferroptosis. O‐GlcNAcylation at the Ser‐555 site of the zinc finger E‐box‐binding protein 1 (ZEB1) increases its stability and nuclear translocation amplifies the transcriptional activity of adipogenesis‐related genes and eventually leads to nephrosis in cells (Figure 5).^136^ The investigation discovered that O‐GlcNAcylation heightens the sensitivity of HCC cells to ferroptosis through Yes‐associated protein (YAP). Furthermore, YAP O‐GlcNAcylation stimulates increased transcription of TFRC, resulting in elevated iron concentrations in HCC cells (Figure 5).^137^ Erastin suppresses the malignant phenotype of hepatoma cells by hindering the O‐GlcNAcylation of c‐Jun, which further represses protein expression, transcriptional activity, and nuclear accumulation of c‐Jun. The authors also noted that c‐Jun's O‐GlcNAcylation regulates GSH synthesis, which displayed a positive correlation with GSH in clinical samples. In summary, O‐GlcNAcyated c‐Jun acts as an obstacle to ferroptosis (Figure 5).^138^

Autophagy is a cellular recycling pathway activated by stress and nutrient deprivation signals. In recent times, an expanding collection of studies has investigated the relationship between autophagy and glycosylation, making it imperative to comprehend the interplay between the two. UNC‐like kinase 1 (ULK1) plays a pivotal role in the initiation of autophagy during starvation. Specifically, during glucose deprivation, ULK1 is O‐GlcNAcylated at the Thr‐754 site by OGT. This O‐GlcNAcylation of ULK1 is critical for the proper activation of VPS34 through ATG14L, which is an essential step in PI(3)P production, phagophore formation, and subsequent initiation of autophagy.^139^ Research indicated that the O‐GlcNAcylation of AMPK diminishes its activity, which in turn inhibits ULK1 activity and autophagy.^140^ OGT may influence the onset of autophagy in cardiomyocytes by enhancing ULK1's activity through O‐GlcNAcylation.^141^

## 4 THE INTERACTION BETWEEN O‐GlcNAcylation AND OTHER PTMs

In this section, we focus on the dynamic interactions between O‐GlcNAcylation and two key PTMs: phosphorylation and ubiquitination, and their roles in regulating protein function. As the kinetic properties of O‐GlcNAcylation become increasingly understood, more evidence suggests that this modification may play a significant role in regulating protein functionality. Specifically, O‐GlcNAcylation can modify S/T residues, which are also sites for phosphorylation, leading to a growing belief in dynamic mutual regulation between O‐GlcNAcylation and phosphorylation. We will introduce early concepts such as the “yin‐yang” hypothesis, supported by examples of proteins like c‐Myc, estrogen receptor (ER)‐β, and endothelial nitric oxide synthase (eNOS). Furthermore, this section will explore the interactions of O‐GlcNAcylation with ubiquitination, particularly examining the ubiquitination processes of OGT and OGA, and how O‐GlcNAcylation stabilizes proteins by inhibiting their ubiquitination. Since O‐GlcNAcylation can influence phosphorylation, and phosphorylation can regulate ubiquitination, O‐GlcNAcylation is conceived to control protein ubiquitination and stability by interacting with phosphorylation. We will delve into the bidirectional effect of O‐GlcNAcylation on protein ubiquitination, analyzing how O‐GlcNAcylation stabilizes proteins by inhibiting ubiquitination and its varying impact on different proteins. Such a thorough viewpoint will facilitate a deeper comprehension of the intricate role that O‐GlcNAcylation plays in protein regulation, along with its possible implications in the progression of diseases and the development of therapeutic strategies.

### 4.1 Phosphorylation

As the kinetic properties of O‐GlcNAcylation have come to light, it has been suggested that this modification may have a regulatory role in protein function. O‐GlcNAcylation can modify Ser and Thr residues, which are also sites of phosphorylation, leading to the increasing belief that there may be dynamic mutual regulation between O‐GlcNAcylation and phosphorylation.^22^, ^142^ Protein phosphorylation is a common biological process that regulates the function and activity of proteins by adding phosphate groups to specific amino acid residues on protein molecules. This modification typically involves transferring phosphate from adenosine triphosphate (ATP) or guanosine triphosphate to specific amino acid residues such as serine, threonine, and tyrosine. Protein phosphorylation plays a crucial role in cell signaling, regulating various biological processes. It is one of the most fundamental and prevalent protein regulation mechanisms within cells and is essential for maintaining normal biological functions in organisms.^143^, ^144^, ^145^ One of the earliest concepts was called the “yin‐yang” hypothesis.^146^ There is a potential for interaction between O‐GlcNAcylation and phosphorylation, indicating the modification of specific residues on proteins by either O‐GlcNAcylation or phosphorylation. Numerous proteins reinforce this idea, including c‐Myc at the Thr‐58 position^147^; ER‐β at Ser‐16, and eNOS at Ser‐1177.^148^ Modifications at proximate sites can also have antagonistic interactions; for instance, histone deacetylase 4 (HDAC4) undergoes O‐GlcNAcylation at Ser‐642, which action impedes the phosphorylation of Ser‐632 mediated by CaMKII.^149^ Certain proteins undergo modifications at distinct sites through both O‐GlcNAcylation and phosphorylation. For example, an increase in Thr‐200 phosphorylation of CaMKIV can reduce O‐GlcNAcylation levels across multiple sites.^150^ There exists an intricate regulatory interplay between kinases and O‐GlcNAcylation. For instance, AMPK can regulate O‐GlcNAcylation levels and can itself be influenced by O‐GlcNAcylation.^151^ GFAT is phosphorylated by AMPK at Ser‐243, reducing GFAT activity and decreasing O‐GlcNAcylation levels.^29^, ^152^ The activation of AMPK leads to enhanced phosphorylation of GFAT and a reduction in O‐GlcNAcylation levels.^153^ AMPK additionally focuses on OGT by phosphorylating it at the Thr‐444 site, which affects only OGT's cellular localization but not its activity. All subunits of AMPK can be targeted by O‐GlcNAcylation.^59^ Therefore, understanding the underlying relationship and interactions between O‐GlcNAcylation and phosphorylation is a crucial

### 4.2 Ubiquitination

O‐GlcNAcylation also interacts with other PTMs.^6^ For instance, O‐GlcNAcylation of OGT and OGA can inhibit protein ubiquitination, thereby suppressing their degradation.^154^, ^155^ O‐GlcNAcylation controls protein ubiquitination via phosphorylation, as it can impact phosphorylation processes,^22^ phosphorylation, in turn, can govern the process of ubiquitination,^156^ it is plausible that O‐GlcNAcylation controls protein ubiquitin and stability through interaction with phosphorylation. Yet, the impact of O‐GlcNAcylation on protein ubiquitination is bidirectional and some studies have found that proteins modified by O‐GlcNAcylation are more stable and not easy to be degraded by ubiquitination. In contrast, some proteins are more likely to be degraded by ubiquitin proteasomes and the specific mechanism remains to be studied. Tumor suppressor protein p53 is intricately regulated, with low levels maintained under normal conditions and rapid accumulation occurring in response to DNA damage through proteasomal degradation.^157^ Administering OGA inhibitors to MCF‐7 cells increases p53 O‐GlcNAcylation, reducing cell survival. O‐GlcNAcylation hinders p53 phosphorylation at Thr‐155 (Ser‐149), decreasing ubiquitination and subsequent degradation. Δ‐lactoferrin acts as a transcription factor, inducing cell cycle arrest by upregulating Skp1 and Bax. Lactoferrin undergoes O‐GlcNAcylation and phosphorylation at Ser‐10.^158^, ^159^ O‐GlcNAcylation enhances lactoferrin stability, maintaining its crucial transcriptional activity. Following activation, lactoferrin undergoes phosphorylation at the Ser‐10 site, promoting transcription, and ultimately undergoes degradation via K‐379 polyubiquitination.^160^ Casein kinase 2 (CK2) is a Ser/Thr PKA linked to cellular proliferation, and DNA repair.^161^ The phosphorylation of the CK2 subunit Thr‐344 enhances protein stability through facilitating interaction with Pin1.^162^ Additionally, O‐GlcNAcylation modifies proximal Ser‐347, counteracting the phosphorylation at Thr‐344 and facilitating its degradation. CRTC2, a transcriptional coactivator regulated by the cAMP response element‐binding protein, serves as a coactivator for the cAMP response molecular binding protein. In the fasting state, glucagon induces the dephosphorylation of CRTC2 at Ser‐171, promoting its translocation into the nucleus to initiate the transcription of genes involved in glycogenesis. Conversely, during feeding, insulin activates the Ser/Thr kinase SIK2, leading to the phosphorylation of CRTC2 at Ser‐171. This phosphorylation causes the relocation of CRTC2 to the cytoplasm, where it undergoes degradation through ubiquitination.^163^ However, whether O‐GlcNAcylation directly affects CRTC2 ubiquitination and protein stability needs to be further studied. A20‐Zinc finger protein inhibits apoptosis and inflammation. Hyperglycemia promotes O‐GlcNAcylation, ubiquitination, and degradation of A20, thereby accelerating atherosclerosis (AS) in diabetic mice.^164^ A20 is a protein that displays both ubiquitin ligase and deubiquitinase activity. Therefore, a plausible hypothesis is that the regulation of A20 protein levels by O‐GlcNAcylation could constitute the tipping point for the subsequent ubiquitination of its target proteins. Keratin pairs 8/18 are expressed in specific combinations during the development and differentiation of tissues. Both keratins are extensively involved in regulating protein interactions, ubiquitination, and filament formation, largely via phosphorylation. Interestingly, keratin 8 and 18 are also highly O‐GlcNAcylated. This modification increases the soluble pool of keratin 8/18, enhances their ubiquitination, and facilitates proteasome‐mediated degradation. Conversely, the deletion of O‐GlcNAcylation sites from keratin 18 makes it more stable. Notably, in vivo, studies have not provided evidence of a correlation between O‐GlcNAcylation and phosphorylation of keratin 8/18.^165^ The molecular chaperone Hsp90 plays a critical role in stabilizing and activating its client proteins. The function of Hsp90 is stringently regulated by cochaperones and various PTMs, which in turn facilitate the activation of its client proteins. The main negative regulator of NRF2 and KEAP was identified as an OGT substrate. The study uncovered that efficient ubiquitination and subsequent degradation of NRF2 require the essential O‐GlcNAcylation of KEAP1 at Ser‐104. Interestingly, variations in glucose levels were observed to parallel changes in both O‐GlcNAcylation levels and NRF2 activation. This connection suggests that O‐GlcNAcylation plays a pivotal role as a crucial link between nutritional sensing and the activation of downstream stress resistance pathways.^166^ In 2021, Huang H found that O‐GlcNAcylation, a newly discovered modification of FOXA2, might boost HCC cell migration by destabilizing FOXA2 and inhibiting the transcriptional activity of its downstream target gene E‐cadherin.^167^

## 5 ROLE OF O‐GLCNACYLATION IN DISEASES

### 5.1 Cardiovascular diseases

The prevalence of CVD has become a substantial menace to human health and welfare. Astonishingly, there has been an almost twofold increase in CVD cases, rising from 271 million in 1990 to 523 million in 2019. Concurrently, cardiovascular‐related fatalities have exhibited a consistent ascent, escalating from 12.1 million to 18.6 million during the same timeframe.^168^, ^169^, ^170^ As a metabolically demanding organ, the heart relies on an adequate energy supply to support its contractile and diastolic functions during both the relaxation and contraction phases. To achieve this goal, the heart requires a significant supply of ATP‐based energy, and CVD results in changes in cellular energy metabolism, stress response, and signaling. However, a common denominator in all of these diseases is the dysregulation of O‐GlcNAcylation signaling. Existing research suggests that the role of O‐GlcNAcylation in CVD is inconsistent. The study primarily focuses on two aspects: on the one hand, O‐GlcNAcylation is believed to have adverse effects on chronic CVD; on the other hand, O‐GlcNAcylation plays a protective role in acute CVD. Therefore, the benefits or drawbacks of O‐GlcNAcylation depend on the particular disease environment.

#### 5.1.1 HF

**FIGURE 6:**
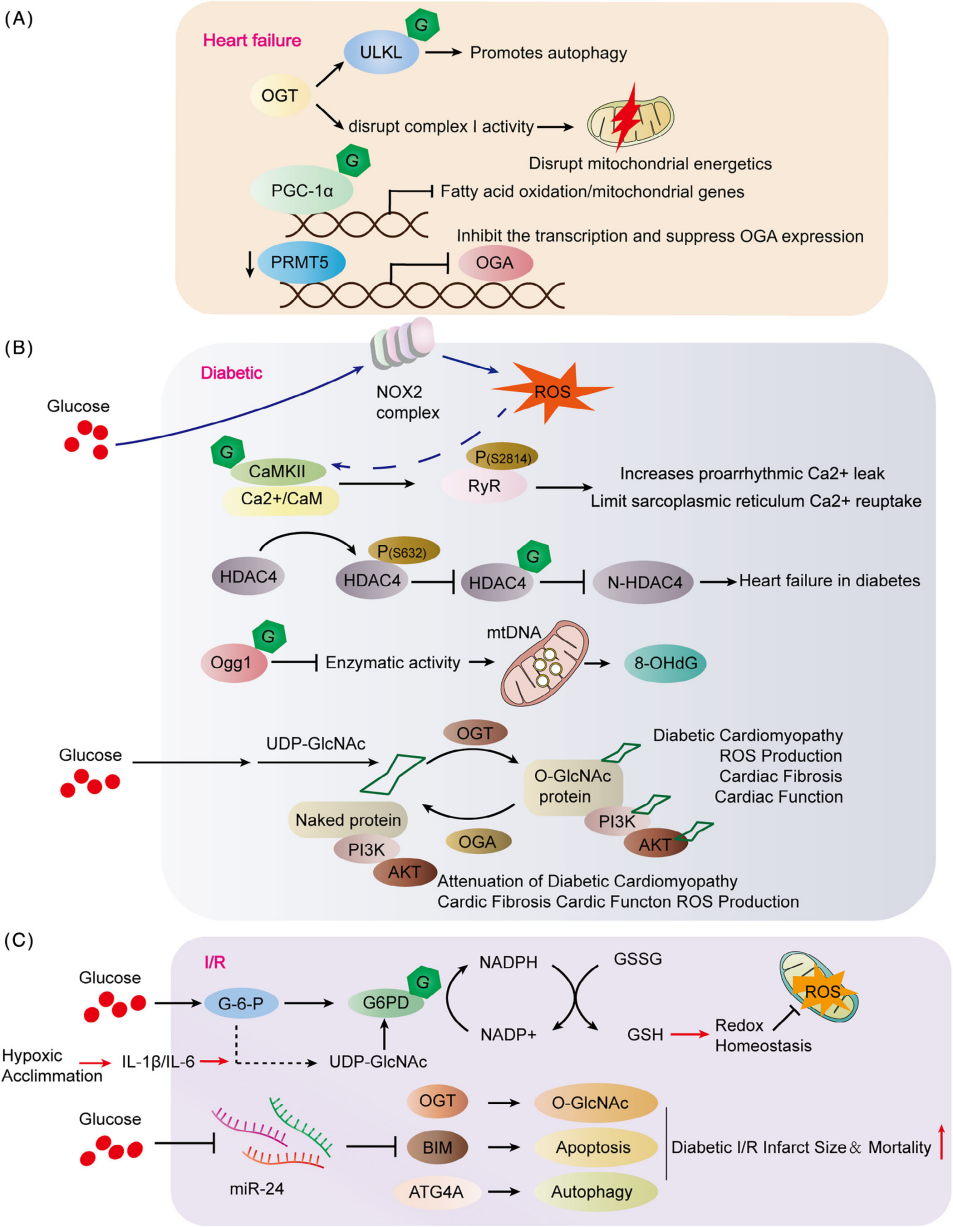
The role of O‐GlcNAcylation in cardiovascular disease. (A) Regulatory mechanisms of HF. Knocking out the OGT gene leads to mitochondrial damage by impacting mitochondrial complex I activity. OGT can alter ULKL's glycosylation modification, thereby affecting early autophagy initiation in cardiomyocytes. The level of O‐GlcNAcylation affects PGC activity, with O‐GlcNAcylation of PGC‐1β inhibiting its activity, expression, and fatty acid oxidation/mitochondrial gene expression, resulting in metabolic dysregulation. DCM hearts exhibit reduced PRMT5 gene expression and PRMT5 deletion inhibits OGA expression by impacting transcriptional activity and triggering abnormal splicing, thereby affecting cardiac function. (B) Regulatory mechanisms of diabetic cardiomyopathy. Ca^2+^/CaM‐dependent protein kinase II‐mediated Ser‐632 phosphorylates HDAC4's N‐terminal fragment while preventing O‐GlcNAcylation of Ser‐642 not only fails to produce the N‐terminal sheet of HDAC4 (protective effect) but also promotes the production of calcium ions. Diabetic hyperglycemia induces acute cardiomyocyte ROS production via NOX2, requiring CaMKIIδ O‐GlcNAcylation at Ser‐280, and CaMKII‐S280 O‐GlcNAcylation increases susceptibility to arrhythmias. O‐GlcNAcylation inhibits Ogg1 activity, leading to mitochondrial DNA lesions. Increased OGT potentially impairs cardiac PI3K(p110α)–Akt signaling. Overexpression of OGA prevents high glucose‐induced mitochondrial respiratory damage in human cardiomyocytes in vitro and protects PI3K(p110α)–Akt signaling in the myocardium of diabetic mice. (C) Regulatory mechanisms of I/R. The upregulation of G6PDH, a rate‐limiting enzyme in the pentose phosphate pathway, enhances cardiac redox homeostasis. Pharmacological inhibition of O‐GlcNAcylation eliminates the effects of hypoxic acclimation (HA) on G6PDH activity, redox balance, and post‐I/R damage in cardiac and cardiomyocytes. miR‐24 targets multiple key proteins, including O‐GlcNAcylation transferase, which protects the myocardium from I/R damage.

The American Heart Association characterizes HF, also referred to as congestive HF, as a syndrome. This is a collection of signs and symptoms that arise due to the heart's inability to pump effectively. HF is described as a chronic, progressively worsening condition where the heart muscle cannot pump sufficient blood to fulfill the body's requirements for blood and oxygen.^171^ In animal models and patients, the hallmark of central muscle failure is an increase in protein O‐GlcNAcylation.^172^ However, it is unclear whether too much O‐GlcNAcylation is a cause or consequence of cardiomyopathy. The researchers found that the hearts of OGT transgenic mice showed increased levels of O‐GlcNAcylation and the potential to develop severe dilated cardiomyopathy (DCM), ventricular arrhythmias, and premature death. In contrast, the O‐GlcNAcylation levels of the hearts of OGA transgenic mice were lower, but the heart function of transgenic mice did not change compared with littermate‐type mice. Even the hearts of OGA transgenic mice have a certain resistance to pathological stress caused by stress overload, and after stress, myocardial O‐GlcNAcylation levels are reduced and pathological hypertrophy is reduced. The experiment also found that although the amount of OGT in the heart muscle of mice is increasing, if OGT is crossed with OGA transgenic mice, cardiomyopathy and premature death can be saved. Transcriptomic and functional studies have also shown damage to the mitochondria of the heart of OGT transgenic mice. OGA transgenic hybridization improves heart damage caused by mitochondrial damage by restoring the activity of complex I. Excess O‐GlcNAcylation can lead to cardiomyopathy, in part because of energy deficiencies. Weakening of O‐GlcNAcylation is beneficial in combating pathological remodeling and HF caused by stress overload (Figure 6).^173^ The study utilized gene knockout techniques to eliminate the OGT gene in cardiomyocytes, resulting in the absence of O‐GlcNAcylation. The findings revealed a notable decrease in autophagy, particularly under fasting conditions. The lack of OGT impacted the early stages of autophagy. The researchers observed O‐GlcNAcylation of Unc‐51‐like autophagy‐activated kinase 1 (ULK1), a crucial kinase initiating autophagy, in cardiomyocytes, with diminished levels in cells where OGT was knocked out. In summary, the study underscores the significance of O‐GlcNAcylation levels in triggering autophagy in cardiomyocytes (Figure 6A; Table 2).^141^

**TABLE 2:** List of selected O‐GlcNAcylation proteins in disease (2010–2023).

Disease	Protein	Protein function	References
Accelerated atherosclerosis and diabetic heart	A20	Decreased A20 expression.	^164^
Heart development	Angiopoietin‐1	Angiopoietin‐1 expression was markedly reduced in OGT cKO hearts. The absence of angiopoietin‐1 in cardiomyocytes resulted in embryonic lethality.	^189^
Tumor‐associated macrophages	Cathepsin B	OGT deletion in macrophages reduces O‐GlcNAcylation and mature cathepsin B levels in the tumor microenvironment (TME), impeding cancer metastasis progression and chemotherapy resistance.	^190^
Hepatocellular carcinoma	YTHDF2 (Ser‐263)	Improved the protein's stability and its cancer‐promoting activity by preventing its ubiquitination.	^191^
Macrophage inflammation	S6K1	Suppress S6K1 phosphorylation and mTORC1 signaling.	^192^
Autoimmune disorders	Foxp3 Stat5	Foxp3 and Stat5 regulate the lineage stability and functionality of regulatory T (Treg) cells, thereby fostering the maintenance of immune homeostasis.	^193^
Alzheimer's disease	Tau	Suppress O‐GlcNAcylation of tau and increase the phosphorylation of tau at specific sites.	^194^

The proteins listed in this table were primarily chosen based on their incorporation within the main content of the article.

Compensatory myocardial hypertrophy may evolve into HF if under conditions of constant stress. It has been reported that protein O‐GlcNAcylation signaling is also associated with myocardial hypertrophy. Peroxisome proliferation activator‐γ‐coactivator‐1α (PGC‐1α) is a transcriptional coactivator that interferes with mitochondrial biosynthesis and is abundantly expressed in myocyte radium.^174^ Moreover, PGC‐1α activity is affected by O‐GlcNAcylation levels. However, a relative decrease in PGC‐1α expression levels has been observed in myocardial hypertrophy, possibly by the fact that PGC‐1β can be directly affected by O‐GlcNAcylation, and its activity and expression are subsequently inhibited, ultimately inhibiting fatty acid oxidation/mitochondrial gene expression, leading to metabolic dysregulation (Figure 6A).^175^ Maintaining cardiac balance involves protein arginine methyltransferase 5 (PRMT5) regulation of reduced protein O‐GlcNAcylation in human DCM hearts. Mice with cardiomyocyte‐specific PRMT5 deletion exhibit DCM and HF, accompanied by increased O‐GlcNAcylation as revealed through transcriptomic and metabolomic analysis. PRMT5 deletion regulates transcription and induces abnormal splicing, inhibiting OGA expression. Remarkably, a positive correlation between PRMT5 and OGA expression was observed in human DCM hearts (Figure 6A).^176^ A variety of research has demonstrated that acute, pharmacologically induced increases in O‐GlcNAcylation levels are effective in reducing cellular and tissue damage. Additionally, these elevations have been shown to promote functional recovery in cardiac tissues.^177^, ^178^, ^179^ Several studies have established a correlation between reduced O‐GlcNAcylation levels and an increased vulnerability of cardiomyocytes to oxidative stress. This suggests that lower levels of O‐GlcNAcylation may compromise the heart cells' ability to withstand oxidative damage.^180^ These studies collectively indicate a growing association between protein O‐GlcNAcylation and cardiovascular events.

#### 5.1.2 Diabetic heart disease

The study findings indicate that elevated OGA expression suppresses p53, resulting in its down‐regulation and subsequently reducing coronary endothelial cell (EC) apoptosis. This sequence of events enhances coronary flow reserve (CFVR) and improves the effect of diabetes on heart function. Moreover, the application of a p53 inhibitor is shown to mitigate coronary EC apoptosis, leading to the restoration of CFVR and cardiac contractility in TH mice.^181^ In vitro and diabetic mouse model studies reveal increased cardioprotective N‐terminal fragments of HDAC4 with elevated O‐GlcNAcylation under high glucose conditions. HDAC4‐deficient mice develop HF in two types of diabetic mouse models., contrasting with wild‐type mice. This underscores HDAC4's crucial role in mitigating diabetes‐induced cardiac damage. Overexpressing HDAC4's N‐terminal fragments prevents HDAC4‐dependent diabetic cardiomyopathy. Mechanistically, O‐GlcNAcylation at Ser‐642 is a vital step in generating these protective fragments, followed by phosphorylation at Ser‐632 by Ca^2+^/CaM‐dependent PKA II. Importantly, O‐GlcNAcylation at Ser‐642 confers cardioprotective benefits in diabetes, counteracting pathological signaling by Ca^2+^/CaM‐dependent PKA II (Figure 6B; Table 2).^149^ Diabetic hyperglycemia induces ROS generation in cardiomyocytes via NADPH oxidase 2 (NOX2) activation, necessitating O‐GlcNAcylation of CaMKII‐δ (Ser‐280). This newly discovered ROS generation mechanism may exacerbate pathological effects of diabetic hyperglycemia, emphasizing a crucial connection between metabolic disruptions and oxidative stress in diabetes (Figure 6B).^182^ Later experimental studies by Bence Hegyi also verified that CaMKII (Ser‐280) O‐GlcNAcylation is required for increased susceptibility to arrhythmias in patients with diabetic hyperglycemia. CaMKII‐dependent phosphorylation of RyR2 (Ser‐2814) significantly increases arrhythmogenic Ca^2+^ leakage, and PLB O‐GlcNAcylation may limit sarcoplasmic reticulum Ca^2+^ reuptake, resulting in impaired excitability‐contraction coupling and arrhythmias in diabetic hyperglycemic patients. In murine animal models, elevated blood sugar levels lead to an upsurge in spontaneous diastolic Ca^2+^ sparks and waves, accompanied by alterations in arrhythmogenic action potentials such as prolonged, alternating, and delayed postdepolarization. These effects are contingent upon the O‐GlcNAcylation of CaMKII at Ser‐280. The action of Ang II relies on NOX2‐mediated oxidation of CaMKII. Diabetes leads to increased Ca^2+^ leakage, phosphorylation of RyR2 Ser‐2814, electrophysiological remodeling, and increased susceptibility to arrhythmias in vivo. Dantrolin reverses CaMKII‐dependent arrhythmogenic RyR‐mediated Ca^2+^ leakage and prevents hyperglycemia‐induced prolonged and delayed postdepolarization of APD (Figure 6B; Table 2).^183^

Elevated oxidative stress‐induced damage to mitochondrial DNA in cardiomyocytes is increasingly recognized as a significant contributor to the development of diabetic cardiomyopathy. This highlights the essential role of mitochondrial integrity and function in preserving cardiac health, especially amid the metabolic disturbances observed in diabetes. A prevalent consequence of mitochondrial DNA damage is the creation of 8‐hydroxy‐2′‐deoxyguanosine (8‐OHdG), capable of inducing mutations if not properly repaired by the O‐GlcNAcylation enzyme 8‐oxo guanine DNA glycosylase (Ogg1). Despite an increase in Ogg1 protein levels, the study revealed significantly lower cardiac Ogg1 activity in mice with type 1 diabetes compared with the comparison group. Diabetic hearts exhibited elevated levels of 8‐OHdG and increased mitochondrial DNA damage. The authors observed high O‐GlcNAcylation of Ogg1 in diabetic mice, which inhibited Ogg1 activity, potentially explaining the accumulation of mitochondrial DNA lesions. The introduction of a dominantly negative O‐GlcNAcylation mutant successfully reduced Ogg1 O‐GlcNAcylation in vivo, restoring Ogg1 enzyme activity, consequently lowering 8‐OHdG levels, and mitigating mitochondrial DNA damage (Figure 6B; Table 2).^184^ Elevated overall protein O‐GlcNAcylation in the hearts of diabetic patients correlates with left ventricular (LV) dysfunction. Investigating the effects of rAAV6‐OGT, rAAV6‐OGA, and an empty carrier (null) in both nondiabetic and diabetic mice, the study revealed that rAAV6‐OGT compromised LV diastolic function and induced maladaptive cardiac remodeling in nondiabetic mice. In contrast, rAAV6‐OGA rescued left ventricular diastolic function and mitigated cardiac remodeling in diabetic mice. The study suggests that the underlying mechanism may involve impaired cardiac PI3K(p110α)–Akt signaling. These findings propose the potential of O‐GlcNAcylation modulators in enhancing cardiac function and mitigating remodeling in diabetic patients (Figure 6B).^185^ A slight elevation in blood pressure (BP) did not significantly affect heart function in either control or high‐fat diet groups. However, in diabetic hearts, this increase led to impaired systolic performance, along with heightened rates of apoptosis and fibrosis. The research pinpointed Beclin‐1 and the antiapoptotic protein (Bcl‐2), both crucial in the early stages of autophagy, as susceptible to O‐GlcNAcylation. In cardiomyocytes derived from type 2 diabetic mice, a delayed autophagic response was observed. This response was partially restored by blocking the entry of glucose into the HBP, which is integral to the synthesis of O‐GlcNAcylation. Acute increases in O‐GlcNAcylation levels in nondiabetic cardiomyocytes can impede autophagy signaling, mimicking the effects of diabetes.^186^ Research has shown that diabetes has harmful impacts on the heart, which may be due to changes in metabolites. Specifically, these effects are caused by a reduction in carbohydrate oxidation and an increased reliance on lipids. Moreover, O‐GlcNAcylation of many components of insulin signaling is common, and high levels can lead to a disruption of insulin signaling. In animal models of diabetes, there is evidence of enhanced lipid metabolism and a corresponding increase in plasma membrane levels of the fatty acid transporter FAT/CD36. The addition of glucosamine to ex vivo perfused hearts leads to a notable increase in cardiac O‐GlcNAcylation levels. This elevation is accompanied by an enhancement in fatty acid oxidation and a rise in the levels of FAT/CD36 in the plasma membrane. This finding underscores the impact of glucosamine on cardiac metabolism, particularly regarding fatty acid utilization.^187^ FAT/CD36 proteins were identified as undergoing O‐GlcNAcylation and were found to be associated with OGT. Additionally, other research has established a connection between O‐GlcNAcylation, fatty acid oxidation, and the translocation of FAT/CD36. This highlights the role of O‐GlcNAcylation in the regulation of lipid metabolism and transport mechanisms.^188^

#### 5.1.3 Ischemia–reperfusion injury

I/R injury is a primary factor in coronary artery disease‐related morbidity and mortality. Three levels of ischemia/reperfusion (I/R)‐induced cardiac damage have been identified depending on the duration of ischemia. Several studies have indicated that I/R damage is strongly associated with the protein O‐GlcNAcylation. Increased protein O‐GlcNAcylation modification can reduce I/R‐induced increase in cardiac oxidative stress and reduce infarct area. G‐6‐PDH, a pivotal enzyme in the pentose phosphate pathway, leads to an increase in the ratios of NADPH/NADP and GSH/GSSG, thereby bolstering cardiac redox homeostasis. The use of pharmacological agents to inhibit O‐GlcNAcylation counteracts the effects of hypoxic acclimation (HA) on G‐6‐PDH activity, redox balance, and the extent of post‐I/R damage in the heart and cardiomyocytes. Interestingly, further enhancement of O‐GlcNAcylation amplifies these benefits, indicating that O‐GlcNAcylation is crucial in mediating the antioxidant and cardioprotective effects induced by HA (Figure 6C).^195^

Myocardial infarction diabetes is also a challenge. The investigators showed that hyperglycemia and hyperinsulinemia in diabetic hearts induced a decrease in microRNA‐24 (miR‐24) and O‐GlcNAcylation led to low survival and increased infarct size due to I/R injury in diabetic myocardium. miR‐24 affects several critical proteins, such as, OGT, which protects the myocardium from I/R damage (Figure 6C).^196^ Neonatal rat ventricular myocytes (NRVMs) are assessed for cell viability, necrosis, apoptosis, and O‐GlcNAcylation levels after exposure to I/R. Cells exposed to glucosamine, hyperglycemia, or the glucosamine derivative carbamate exhibited elevated O‐GlcNAcylation levels and improved cell viability post‐I/R compared with untreated cells. Azazexerin, inhibiting glucose metabolism through the HBP, mitigated the enhancement in survival under hyperglycemic conditions. Reperfusion without sugar reduced O‐GlcNAcylation levels and reduced cell viability compared with normal glucose conditions. The effects of glucosamine and PUGNAc on cell viability are associated with decreased calcineurin activation, indicating that elevated O‐GlcNAcylation can diminish I/R‐induced cytosolic Ca2+ raise.^197^ The treatment approach of glucose–insulin–potassium (GIK) therapy is commonly employed for I/R injury, and the O‐GlcNAcylation induced by GIK is seen as a potential mechanism for its cardioprotective effects. Normally, insulin plays a beneficial role by promoting the phosphorylation of Akt and activating Akt‐mediated cell survival signaling pathways.^198^ Elevated O‐GlcNAcylation in obesity competitively inhibits insulin‐induced phosphorylation of Akt, thereby impairing Akt activation. This interference results in diminished cell proliferation and reduces the protective effects of insulin against I/R injury.^199^ Yet, the current comprehension implies a paradoxical nature to the influence of protein O‐GlcNAcylation. While a moderate increase in O‐GlcNAcylation can alleviate damage from I/R in the heart, excessively elevated levels may diminish the advantageous effects of GIK therapy. As a result, controlling the extent of O‐GlcNAcylation in the body has become progressively vital.

#### 5.1.4 Atherosclerosis

AS, a type of arteriosclerosis, involves the buildup of substances like fat and cholesterol on the walls of arteries, resulting in the formation of plaques. This buildup of plaque can narrow the arteries and obstruct blood flow, and if the plaque ruptures it can cause the formation of blood clots. Although AS is often associated with heart disease, recent research studies have discovered that it is strongly linked to the protein O‐GlcNAcylation. This insight is particularly pertinent as accelerated AS stands as the primary cause of morbidity and mortality in individuals with diabetes. Hyperglycemia, an established contributing factor for both diabetes and AS, is noted to decrease the expression levels of the A20 protein. Intriguingly, despite this decrease in protein levels, The mRNA levels of A20 remain unaffected or increased in ECs and smooth muscle cells (SMC) cultured in high glucose conditions, a scenario associated with O‐GlcNAcylation and ubiquitination of A20. These findings suggest that reinstating A20 levels through inhibiting O‐GlcNAcylation, blocking proteasome activity, or enhancing A20 expression could potentially prevent the upregulation of late O‐GlcNAcylation end products, such as RAGE receptors and PKCβII phosphorylation. These molecules are primary triggers for atherosclerotic signaling in EC/SMC cells exposed to high glucose concentrations.^164^ Hyperglycemia/hyperglycemia regulates vascular A20 expression through O‐GlcNAcylation‐dependent ubiquitination and proteasome degradation. This may be the key to the accelerated pathogenesis of AS in diabetes. Abnormal O‐GlcNAcylation of intracellular proteins has also been validated as possibly associated with glucose toxicity to vascular tissue (Table 2).^200^ Hyaluronic acid accumulation in arterial walls is associated with the development of conditions like AS and restenosis. This is due to changes in the microenvironment in the vascular wall tissue, which plays a critical role in the formation of vascular lesions and other CVDs.

#### 5.1.5 Hypertension

Elevated arterial BP can be ascribed to a variety of factors, including modifications in cardiac output and harm to target organs.^201^ Remarkably, the well‐established mechanisms that can modify BP and cardiac function are also influenced by O‐GlcNAcylation.^202^ Excessive and persistent O‐GlcNAcylation has been observed in both human and animal models with heart muscle dysfunction, heart remodeling, aortic constriction, and hypertension.^203^ In Zucker (Type 2 Diabetes) rats, elevated blood sugar cause an increase in O‐GlcNAcylation, a reduction in cardiac muscle cell calcium spikes, and an extended period of diastole, leading to compromised heart muscle contraction and prolonged diastolic duration.^204^ In models of hypertension, there is a significant increase in systolic BP, cardiac muscle thickening, and enhanced protein O‐GlcNAcylation in the LV. This research suggests that persistent hypertension and aortic constriction lead to an elevation in O‐GlcNAcylation, which correlates with increased OGT protein levels, indicating the significance of OGT in cardiac protein O‐GlcNAcylation. In LV biopsy specimens from patients with severe aortic valve narrowing, O‐GlcNAcylation levels were 65% higher than in control subjects. Additionally, studies have shown a link between heightened O‐GlcNAcylation and GFAT expression in SHRs. Research found that adult SHRs with hypertension showed elevated O‐GlcNAcylation in the cortical region of the kidney, hinting that alterations in proteins of the proximal tubules relate to increased O‐GlcNAcylation in the adult SHR renal cortex. However, young SHRs did not show changes in O‐GlcNAcylation levels or BP, implying that a rise in cortical O‐GlcNAcylation might be linked to the onset of hypertension. Supporting this, GFAT inhibitors have been observed to decrease overall O‐GlcNAcylation in SHR and significantly reduce BP.^205^ The administration of deoxycorticosterone acetate salt elevated ET‐1 levels and induced increased BP in hypertensive rats by upregulating vascular GFAT expression and promoting O‐GlcNAcylation.^206^, ^207^

#### 5.1.6 Hypoplastic heart

O‐GlcNAc is widely regarded as a crucial nutritional sensor. To gain a deeper understanding of its role in the early stages of life, researchers conducted a detailed assessment of the levels of protein O‐GlcNAc, associated regulatory enzymes, and changes in metabolites. They further explored the specific impact of O‐GlcNAcylation on cardiac proteins. Notably, the study found that the levels of protein O‐GlcNAc in the heart significantly decreased from birth to adulthood. In contrast, the trends in the liver and brain were the exact opposite of those observed in the heart. It is worth noting that the levels of O‐GlcNAc were not directly influenced by weaning diets. Additionally, the expression of enzymes and metabolites regulating O‐GlcNAc exhibited distinct specificity in different tissues. Through mass spectrometry analysis, researchers successfully identified O‐GlcNAc proteins in the heart, which primarily participate in stress responses and energy metabolism processes.^208^ This research indicates that protein O‐GlcNAc levels are not directly tied to dietary intake and vary during postnatal development based on timing and tissue type. Mass spectrometry identified potential proteins with unique O‐GlcNAc features crucial for biological development. Lack of OGT in early fetal cardiomyocytes results in multiple cardiac defects. Notably, in mice with cardiomyocyte‐specific OGT knockout, there is a significant decrease in angiopoietin‐1, vital for heart and coronary artery development. Thus, OGT is essential in cardiac development by regulating angiopoietin‐1 expression in cardiomyocytes (Table 2). The study emphasizes the significance of each component in cardiac development for the heart's proper formation and function.^189^

### 5.2 Cancer

Cancer is the second leading cause of death after CVD worldwide, representing a remarkable threat to humans’ lives and health. According to the latest data from the International Agency for Research, nearly 19.3 million new cancer diagnoses and over 10.0 million deaths occurred in 2020.^209^ Thus, it is urgent to clarify the underlying mechanisms involved in cancer progression and to identify novel therapeutic targets for cancer clinical treatment.

Many types of tumor cells exhibit elevated levels of O‐GlcNAcylation, potentially due to increased nutrient flux and metabolic alterations in these cells. Additionally, O‐GlcNAcylation can modulate key proteins associated with cell cycle and proliferation, such as p53, c‐Myc, and AKT, affecting their stability, activity, and subcellular localization. It also regulates pathways related to cell apoptosis and survival, influencing tumor cell viability. In summary, the relationship between O‐GlcNAcylation and cancer is intricate, with this PTM playing a pivotal role in tumor onset and progression.^210^ Therapeutic strategies targeting O‐GlcNAcylation may offer novel opportunities for cancer treatment. The study explores the role of glucose metabolism in enhancing the protumor functions of tumor‐associated macrophages (TAMs). Increased glucose uptake in M2‐type TAMs activates O‐GlcNAcylation, promoting cancer metastasis and resistance to chemotherapy. This process increases the levels of the enzyme cathepsin B in the tumor environment, facilitated by OGT. A lack of OGT in macrophages reduces cancer spread and chemoresistance. In humans, high OGT levels in TAMs are linked to cathepsin B expression, predicting chemotherapy results and cancer outcomes. This research highlights the importance of glucose metabolism in tumor‐promoting TAMs and provides insights into the mechanisms involved (Table 2).^190^ The study shows that breast cancer reduces O‐GlcNAc protein modification in skeletal muscles through miR‐122 contained in extracellular vesicles. This affects muscle protein balance and function. O‐GlcNAcylation interacts with NEK10‐mediated phosphorylation of RYR1, causing its increased degradation. Breast cancer‐driven miR‐122 reduces OGT, raising RYR1 levels. Muscle O‐GlcNAcylation is also influenced by hypoxia and lactate, especially after exercise. In cancer, reduced O‐GlcNAcylation boosts cytosolic Ca^2+^ leading to muscle damage. This is linked to decreased muscle mass and function in mice with tumors, highlighting O‐GlcNAcylation's importance in muscle health.^211^ Research reveals that under high glucose conditions, O‐GlcNAcylation levels and ferroptosis significantly increase in mesenchymal pancreatic cancer cells. Specifically, ZEB1's O‐GlcNAcylation plays a pivotal role. Mechanistically, glucose‐induced O‐GlcNAcylation of ZEB1 at Ser‐555 enhances its stability and nuclear translocation, subsequently upregulating lipid synthesis genes FASN and FADS2, leading to lipid peroxidation‐dependent ferroptosis (Table 2). These findings highlight a novel role of glucose metabolism and O‐GlcNAcylation in ferroptosis sensitivity, offering potential therapeutic avenues for refractory tumors.^136^ The study reveals that HBV infection notably elevates O‐GlcNAcylation of the RNA m6A reader YTH N6‐methyladenosine RNA binding protein 2 (YTHDF2). O‐GlcNAcylation of YTHDF2 at Ser‐263 enhances its protein stability and cancer‐promoting activity (Table 2). In terms of mechanism, YTHDF2 stabilizes the transcription of MCM2 and MCM5, facilitating the progression of the cell cycle and contributing to tumorigenesis in HBV‐associated HCC.^191^ Aberrant O‐GlcNAc modifications correlate with cancer. The study emphasizes O‐GlcNAcylation's role in cancer metabolism and epigenetics, impacting signaling proteins, metabolic enzymes, and transcription factors, particularly in chromatin interactions, underscoring its regulatory significance in cancer progression.

### 5.3 Inflammation

Inflammation is a reaction of the body to harm or infection, involving a variety of cells and molecules. Prolonged, mild, chronic inflammation characterizes the progression of obesity and type 2 diabetes. This inflammatory condition is associated with many subsequent health issues, including AS, insulin resistance, and an increased risk of autoimmunity. All these problems are linked to overnutrition and elevated body fat.^212^, ^213^, ^214^, ^215^ Under high‐glucose conditions, O‐GlcNAcylation can enhance the transcriptional activity of NF‐κB, leading to the development of inflammation‐related complications in diabetes. Conversely, in situations like acute cardiovascular injury, O‐GlcNAc can inhibit NF‐κB transcriptional activity, exerting anti‐inflammatory functions. This indicates that O‐GlcNAcylation is a double‐edged sword in inflammatory diseases. In other literature, O‐GlcNAcylation has also been shown to alter the function of key transcription factor regulatory elements, such as STAT and others.^189^, ^216^, ^217^ O‐GlcNAcylation regulates these factors, impacting their stability, function, and movement into the nucleus, and thus influencing their capacity to manage the inflammatory response. The research emphasizes that while increased glucose metabolism in immune cells is a characteristic feature of many inflammatory conditions, the precise role of glucose metabolic pathways in inflammation remains somewhat unclear. The study identified an anti‐inflammatory effect associated with O‐GlcNAc signaling in connection to the HBP. In LPS‐activated macrophages, despite increased glycolysis, HBP activity and protein O‐GlcNAcylation were reduced. Depletion of OGT, a key enzyme for protein O‐GlcNAcylation, heightened the immune response and worsened inflammation related to sepsis. At the molecular level, OGT‐induced O‐GlcNAcylation on the RIPK3 kinase restricted specific RIPK3 interactions, thus dampening innate immune and necroptotic signaling. Overall, the study underscores the crucial interplay between glucose metabolism and the activation of immune cells in the context of sepsis‐related inflammation (Table 2).^218^ The study uncovers that an excess of nutrients triggers an upsurge in O‐GlcNAc pathway within macrophages. During the proinflammatory activation of macrophages, O‐GlcNAc pathway is decreased. Disrupting O‐GlcNAc pathway by eliminating OGT boosts the proinflammatory polarization of macrophages, induces inflammation and lipolysis in adipose tissue, raises lipid accumulation in peripheral tissues, and worsens tissue‐specific and systemic insulin resistance in mice subjected to a high‐fat diet. OGT influences macrophage proinflammatory activation by facilitating the O‐GlcNAcylation of ribosomal protein S6 kinase beta‐1 (S6K1), hindering its phosphorylation, and suppressing mTORC1 signaling (Table 2).^192^ The study reveals that O‐GlcNAc posttranscriptional modifications upon T‐cell receptor activation stabilize Forkhead box protein P3 (FOXP3) protein and activate signal transducer and activator of transcription 5 (STAT5), integrating these crucial pathways. While Treg cells with O‐GlcNAc deficiencies develop normally, they exhibit slightly reduced FOXP3 expression, compromised lineage stability, and functional efficacy, leading to lethal autoimmune diseases in mice. Additionally, the absence of protein O‐GlcNAcylation weakens the IL‐2/STAT5 signaling. Overall, these findings underscore the essential role of protein O‐GlcNAcylation in the stability and functional efficacy of Treg cells.^193^ The study delved into the function of enzymes related to O‐GlcNAcylation in the gut microbiome. It found that bacterial OGAs and OGTs, especially in the Bacteroidetes and Firmicutes phyla, are abundant in healthy guts but decrease in those with ulcerative colitis. In laboratory tests, bacterial OGAs were found to break down host O‐GlcNAcylated proteins, including a key component for NF‐κB activation. In animal tests, these gut‐derived OGAs protected mice from chemically induced colitis. The research uncovers a new enzymatic activity in the gut microbiome and suggests bacterial OGAs as a potential treatment for colitis.^219^ The aforementioned studies reveal the pivotal role of O‐GlcNAcylation in various physiological and pathological processes, including inflammation, immune cell activation, obesity‐induced insulin resistance, and gut microbiome functions. These findings emphasize the significance of O‐GlcNAcylation in regulating inflammatory responses, immune homeostasis, and gut health, offering novel therapeutic strategies for related diseases.

### 5.4 Nervous system

The brain, as one of the organs in the body with a high energy demand, requires a significant amount of glucose. However, the brain is unable to store glucose. Consequently, the levels of glucose in peripheral blood can potentially affect the normal functioning of the brain.^220^, ^221^ Studies have already demonstrated that when there is an abnormal uptake of glucose in the brain, brain diseases may ensue.^222^ Therefore, the HBP, acting as a nutritional sensor, has become a key focus in the research on the development of brain diseases.

#### 5.4.1 Alzheimer's disease

Alzheimer's disease (AD), a prevalent cause of dementia, primarily afflicts the elderly, with up to 70% of dementia cases. Patients experience memory loss and distressing neuropsychiatric symptoms, including agitation, aggressiveness, hallucinations, and mood disturbances. With an aging population, AD is expected to affect around 130 million people by 2050.^222^ As the cases of AD continue to rise, the demand for care facilities and personnel is steadily increasing. The lack of effective treatment for the disease has led to an increase in AD‐related mortality rates, underscoring the pressing need for research into its pathophysiological mechanisms and targeted therapies. In AD, regions of the brain prone to neurodegenerative changes exhibit reduced glucose metabolism. The ability of O‐GlcNAc to respond to changes in cellular glucose levels positions it as a potential factor in neurodegenerative processes. O‐GlcNAcylation has the ability to block necrotic apoptosis, providing a protective effect in AD. In models of AD, heightened necroptosis has been detected, but the O‐GlcNAcylation of RIPK3 in 5xFAD mice models (transgenic mice with five familial AD) led to its reduction.^223^ Enhanced O‐GlcNAcylation ameliorates AD symptoms, reducing Aβ burden and neuroinflammation, repairing damaged mitochondria, and restoring microglial function. Experiments on SH‐SY5Y cells revealed that inhibiting OGA increased ERK1/2 phosphorylation. Disruption of O‐GlcNAc homeostasis intensifies ERK signaling in AD.^224^ Tubulin‐associated unit (Tau) protein is extensively O‐GlcNAcylated in the human brain, but the level of O‐GlcNAcylation of tau is reduced in the brains of AD patients. Tau protein's O‐GlcNAcylation is extensive in the human brain but diminished in AD. The capacity of NAD‐dependent deacetylase sirtuin‐1 (SIRT1) to deacetylate CREB, thereby reducing OGT expression, influences tau protein's O‐GlcNAcylation, suggesting SIRT1 is a potential therapeutic target for tau‐associated disorders (Table 2).^194^ The development of PET tracers, like BIO‐735 and BIO‐578, is crucial for therapeutic OGA inhibitors. BIO‐578, in particular, shows promise for human studies due to its reversible binding kinetics.^225^ BIO‐578 tracer is promising for further human characterization. In essence, the research underscores the intricate role of O‐GlcNAcylation in AD and its potential as a therapeutic target.

#### 5.4.2 Parkinson's disease

Parkinson's disease (PD) is the second most common neurodegenerative disorder worldwide, with an increased incidence in older age, and a higher prevalence in men than in women. Both motor and nonmotor symptoms constitute the majority of the clinical features of PD. Patients with PD exhibit a variety of symptoms that impact normal life, such as bradykinesia, muscle rigidity, and gait difficulties. Research has found that the postmortem brains of PD patients show an increase in O‐GlcNAcylated proteins, indicating that O‐GlcNAcylation plays a significant role in PD. The brainstem's dopamine system, critical for intentional movement and decision‐making, is limited in neuron count but wide‐ranging in its functions, and it is particularly vulnerable to PD. Investigations have shown the importance of O‐GlcNAcylation in maintaining the standard functions of dopamine neurons, with its dual‐direction adjustment crucially influencing these neurons on various levels, including molecular, synaptic, cellular, and behavioral aspects. Importantly, interventions that increase O‐GlcNAcylation, either through genetics or pharmacology, have been shown to mitigate the effects of neurodegeneration, synaptic damage, and motor disorders in PD animal models.^226^, ^227^ α‐Synuclein, a protein linked to synucleinopathies and neurodegenerative conditions, forms harmful aggregates. The targeted modification of this protein at the Ser‐27 site by O‐GlcNAcylation effectively reduces its tendency to aggregate, while maintaining its interactions with cellular membranes and its structural flexibility. Furthermore, O‐GlcNAcylation alters the phosphorylation process of α‐synuclein in lab conditions and inhibits its harmful effects when introduced externally into cell cultures.^228^ In a related study, it was observed that α‐synuclein is modified by O‐GlcNAcylation at nine distinct positions in proteomic analyses conducted on both mouse and human tissues. This modification could potentially impact the protein's tendency to aggregate and may become a crucial factor and potential treatment target in neurodegenerative disorders. The researchers created six α‐synuclein variants with specific O‐GlcNAc modifications and found through various biochemical tests that O‐GlcNAc generally reduces the aggregation of α‐synuclein and can also modify the structure of its aggregates. Additionally, a version of α‐synuclein with three O‐GlcNAc modifications was found to suppress the aggregation of the protein in its natural, unmodified form.^229^ These insights indicate that enhancing O‐GlcNAcylation may decelerate the advancement of synucleinopathies, reinforcing the overarching role of O‐GlcNAc in averting the aggregation of proteins. These research outcomes are significant for understanding the mechanisms behind the development of PD and related neurodegenerative disorders. They not only uncover the potential role of O‐GlcNAcylation in neurodegenerative diseases, particularly PD but also provide a scientific foundation for the development of new therapeutic strategies.

## 6 O‐GLCNACYLATION AS A POTENTIAL THERAPEUTIC TARGET

Scientists are actively exploring how to treat various diseases such as cancer, heart disease, diabetes, and neurodegenerative diseases by regulating O‐GlcNAcylation. The focus of research is on developing small molecule inhibitors that can precisely regulate OGT and OGA. These enzymes significantly impact cellular functions by adding or removing O‐GlcNAc modifications on cellular proteins.^249^ OGT, a key glycosyltransferase closely associated with various cellular functions, can specifically target certain proteins through interactions with adaptor or scaffold proteins. For example, in the liver, OGT's action under fasting conditions involves interaction with HCF1, localizing to PGC‐1α, increasing its O‐GlcNAcylation, and thereby enhancing the stability of PGC‐1α and upregulating gluconeogenic genes.^125^ OGT also interacts with other proteins such as p38 MAPK, REV‐ERBα, and OGA, possibly forming an “O‐GlcNAczyme” complex consistent with the rapid and reversible changes in O‐GlcNAcylation.^65^, ^250^, ^251^ However, knockout of the OGT gene in higher animal models leads to embryonic lethality, limiting its study as a potential therapeutic target.^252^, ^253^ Therefore, developing effective selective OGT inhibitors is crucial for understanding the processes of O‐GlcNAcylation and the creation of associated pharmaceuticals. Discovered inhibitors of OGT encompass substrate analogs, bisubstrate inhibitors, and those identified through high‐throughput screening, but they generally suffer from insufficient specificity and poor cellular permeability.^249^ The active site of OGT is hydrophilic, accommodating various peptide sequences, with substrate selectivity determined not by specific contacts of OGT side chains but by protein interaction with the TPR domain. Substrate analogs like alloxan and UDP‐5S‐GlcNAc, although effective in occupying the OGT substrate‐binding site, do not cause a global decrease in O‐GlcNAcylation levels in cells, possibly due to lack of cellular permeability.^238^, ^254^ Researchers have addressed this issue by “hijacking” the HBP, using compounds like UDP‐5S‐GlcNAc. There are also bisubstrate inhibitors like Goblin1 and Goblin2, which replace the GlcNAc part of the donor substrate with a short linker and covalently connect to the acceptor peptide, but they lack cellular permeability. Therefore, future research needs to explore new, efficient, and highly selective OGT inhibitors to better understand the regulatory mechanisms of O‐GlcNAcylation and support drug development. The development of OGA inhibitors is also an important area in the study and therapeutic application of O‐GlcNAc functions. PUGNAc is one of the earliest and most widely used OGA inhibitors, but its structural design leads to selectivity issues with other glycoside hydrolases.^249^, ^255^ To address this issue, based on PUGNAc and its crystal structure with OGA, highly efficient and very selective OGA glucosyl imidazole derivatives like GlcNAcstatin, especially GlcNAcstatin G, have been developed, showing low nanomolar level inhibition effects and extremely high selectivity.^256^, ^257^ Another OGA inhibitor, NAG‐thiazoline, is effective but not highly selective, leading to the development of derivatives like NButGT and Thiamet G, the latter showing very high potency and selectivity.^258^, ^259^, ^260^, ^261^ Thiamet G has shown potential in AD mouse models by increasing O‐GlcNAc levels to slow neurodegenerative changes.^262^ Furthermore, further optimization of the Thiamet G structure, such as the development of MK‐8719, has shown improved drug‐like properties and has entered clinical trials.^263^ Overall, the development of OGT and OGA inhibitors is a significant advancement in the study and treatment of diseases related to O‐GlcNAcylation. Although the currently available inhibitors have problems with specificity and cellular permeability, researchers are continuously innovating to overcome these obstacles. Particularly, the development of OGA inhibitors like GlcNAcstatin G and Thiamet G has shown great potential in treating neurodegenerative diseases, diabetes, and cancer. With more efficient and selective OGT and OGA inhibitors in development, a multitude of effective inhibitors are emerging, We have summarized the findings (Table 3). future treatments in this field may become more precise and effective, bringing new hope to patients.

**TABLE 3:** Inhibitors used to study O‐GlcNAc levels in cells and tissues.

Inhibitors	Characteristics	Constraint	References
Azaserine	Azaserine functions as a purine antagonist and exhibits a structural resemblance to glutamine. Its mode of action involves competitively inhibiting GFAT, a pivotal enzyme in the metabolism of glutamine.	Absence of selectivity	^230^, ^231^, ^232^, ^233^
6‐Diazo‐5‐oxo‐l‐norleucine (DON)	DON is employed as an inhibitor for GFAT. Given its resemblance to glutamine, it can access the catalytic centers of these enzymes and impede their activity through covalent binding, or more precisely, through the process of alkylation. Undergoing clinical trials in combination with recombinant glutaminase for the treatment of diverse solid tumors.	Absence of selectivity	^234^, ^235^, ^236^
Alloxan	A mild inhibitor of OGT.	At high doses, alloxan exhibits toxicity to the liver and kidneys.	^237^
5‐Thioglucosamine (5SGlcNAc)	5SGlcNAc is converted to UDP‐5S‐GlcNAc intracellularly, inhibiting the function of OGT.	UDP‐5S‐GlcNAc exhibits cell line‐specificity for OGT.	^238^, ^239^, ^240^
OSMI	Forms a low nanomolar affinity interaction with OGT.	Exhibits cell permeability.	^241^, ^242^, ^243^, ^244^
ST045849 (TT04)	Inhibiting the function of OGT.	The specificity of OGT is unclear.	^245^
PUGNAc	Used as an inhibitor of OGA.	Poor water solubility, sensitive to air, light, heat, and temperature.	^246^, ^247^, ^248^

The inhibitors listed in this table were primarily chosen based on their incorporation within the main content of the article.

O‐GlcNAcylation, as a key intracellular regulatory mechanism, offers a new pathway for treating a variety of diseases but also has certain advantages and limitations. This mechanism involves various cellular processes, such as signal transduction, transcriptional regulation, and protein stability, thus showing great potential in treating diseases like neurodegenerative diseases, cancer, and heart disease. Particularly in neurodegenerative diseases, regulating O‐GlcNAcylation may positively impact the survival and function of nerve cells, thereby slowing disease progression. Since O‐GlcNAcylation affects a variety of proteins and cellular pathways, it provides multiple potential therapeutic targets. However, the application of this mechanism also faces challenges. The first is the challenge of specificity: Since O‐GlcNAcylation plays a role in many cellular processes, it becomes very difficult to precisely target specific diseases without affecting other important functions. Additionally, the dynamic and complex nature of O‐GlcNAcylation makes developing drugs with specificity and efficacy a challenge. Currently, available OGT and OGA inhibitors suffer from insufficient specificity and poor cellular permeability. Furthermore, drugs regulating O‐GlcNAcylation might cause undesirable side effects due to its role in multiple cellular processes. At the same time, little is known about the effects and safety of long‐term regulation of O‐GlcNAcylation, requiring more research to elucidate. In summary, O‐GlcNAcylation as a treatment method offers new perspectives and possibilities, but also faces a series of challenges such as specificity and safety. Future research needs to focus on resolving these limitations to develop more effective and safe treatment schemes.

## 7 CONCLUSION

Since its initial identification, the exploration of O‐GlcNAcylation has undergone significant progress. While early investigations primarily focused on its associations with diabetes and heightened blood glucose levels, it has now evolved into a widely recognized PTM that profoundly shapes cellular regulation. This modification is implicated in a diverse array of disorders, including cancers and CVDs. Despite substantial strides in comprehending O‐GlcNAcylation, there is still a considerable amount to uncover. It has become increasingly apparent that O‐GlcNAcylation plays a crucial role in the functionality of various organ systems, providing insights into the complexities of numerous signaling pathways. Moreover, state‐of‐the‐art research methodologies are offering fresh perspectives on how O‐GlcNAcylation influences various ailments and contributes to the advancement of these conditions.

With recent advancements in molecular and pharmacological methodologies, our exploration into the essential physiological functions of O‐GlcNAcylation in proteins and its impact on various diseases has deepened. This makes the ongoing study of O‐GlcNAcylation an active and fruitful field, holding the promise of uncovering new treatments for diverse health conditions. Gaining a comprehensive understanding of this modification is crucial for further progress in this area. Initially, the use of radiolabeling through biochemical techniques was instrumental in detecting monosaccharide modifications on cellular proteins. Although structurally simple, the precise detection of O‐GlcNAc has always been rather complex. Nowadays, chromatography, spectroscopy, and biochemical analyses are capable of dissecting the factors involved in the O‐GlcNAc cycle. However, analytical challenges persist and have yet to be fully addressed.^264^, ^265^ The past decades have witnessed explosive growth in tools for O‐GlcNAc research.^266^ For example, a series of antibodies have become effective tools for detecting O‐GlcNAc.^267^, ^268^, ^269^, ^270^ Furthermore, metabolic biomarkers can also identify O‐GlcNAc levels. The enzyme inhibitors mentioned earlier assist in a better understanding of the key enzyme catalytic processes within the O‐GlcNAc cycle. With the development of new technologies and instruments, it is anticipated that this research field will continue to accelerate.

In clinical applications, biomarkers are widely employed to identify the occurrence and progression of diseases, as well as to assess the effectiveness of treatment measures. Additionally, the detection of biomarkers allows for the prediction of a patient's vulnerability to specific diseases and their response to drug therapy at various stages of the disease. Despite the existence of different biomarkers for various diseases, the demand for sensitive and accurate biomarkers remains crucial.^271^ The preceding data reveals the critical role of O‐GlcNAcylation in metabolic disruptions; hence, the discovery of biomarkers targeting O‐GlcNAcylation is of paramount importance. OGT stands out as a primary candidate for biomarker development. Interestingly, the substantial increase in OGT levels in cancer cells makes it an effective indicator for cancer monitoring. Researchers have employed immunohistochemistry to demonstrate that in cholangiocarcinoma tissue samples, both OGT and overall O‐GlcNAcylation levels exhibit significant elevation, while OGA levels are concurrently found to be reduced.^272^ Moreover, high OGT expression correlates with poorer survival rates in cholangiocarcinoma patients, suggesting its potential as a valuable predictive marker for cholangiocarcinoma.^273^ In comparison with OGT, OGA has received relatively less attention. However, new evidence suggests that OGA might also serve as a potent biomarker. For instance, a study by Zhu et al.^274^ evaluated the levels of OGT, OGA, and O‐GlcNAcylation in liver tissues from healthy controls, HCC patients, and HCC patients who underwent liver transplantation. Their research highlighted a significant elevation in overall O‐GlcNAcylation levels in HCC tissues compared with normal liver tissues. Furthermore, a notable increase in overall O‐GlcNAcylation was observed in recurrent HCC tissues. These findings strongly suggest a correlation between O‐GlcNAcylation levels, the development of HCC, and the recurrence of tumors following liver transplantation. Remarkably, levels of OGA were markedly lower in recurrent HCC specimens compared with the majority of nonrecurrent HCC cases, while OGT levels did not show a correlation with HCC recurrence posttransplantation. Consequently, OGA emerges as a potential independent predictive marker for tumor recurrence in HCC. Although various diagnostic methods are currently employed, research in this domain remains somewhat limited. Nevertheless, given its profound scientific, clinical, and commercial significance, substantial growth in research in this area is anticipated in the near future.

## AUTHOR CONTRIBUTION

All the authors contributed significantly to this work. Jianxun Wang and Xiang Ao provided direction and guidance throughout the preparation of this manuscript. Lin Ye, Wei Ding, Yi Jia, Dandan Xiao, and Zhonghao Zhao collected and prepared the related literature. Lin Ye and Wei Ding drafted the manuscript. Lin Ye and Xiang Ao reviewed and made significant revisions to the manuscript. All authors have read and approved the final manuscript.

## CONFLICT OF INTEREST STATEMENT

The authors confirm that there are no conflict of interest.

## ETHICS STATEMENT

Not applicable.

## Data Availability

Not applicable.

## References

[1] Zhong Q , Xiao X , Qiu Y , et al. Protein posttranslational modifications in health and diseases: functions, regulatory mechanisms, and therapeutic implications. MedComm. 2023;4(3):e261. 37143582 10.1002/mco2.261 PMC10152985

[2] Wu X , Xu M , Geng M , et al. Targeting protein modifications in metabolic diseases: molecular mechanisms and targeted therapies. Signal Transduct Target Ther. 2023;8(1):220. 37244925 10.1038/s41392-023-01439-y PMC10224996

[3] Lee JM , Hammarén HM , Savitski MM , Baek SH . Control of protein stability by post‐translational modifications. Nat Commun. 2023;14(1):201. 36639369 10.1038/s41467-023-35795-8 PMC9839724

[4] Vu LD , Gevaert K , De Smet I . Protein language: post‐translational modifications talking to each other. Trends Plant Sci. 2018;23(12):1068‐1080. 30279071 10.1016/j.tplants.2018.09.004

[5] Xu P , Cai X , Guan X , Xie W . Sulfoconjugation of protein peptides and glycoproteins in physiology and diseases. Pharmacol Ther. 2023;251:108540. 37777160 10.1016/j.pharmthera.2023.108540 PMC10842354

[6] Yang X , Qian K . Protein O‐GlcNAcylation: emerging mechanisms and functions. Nat Rev Mol Cell Bio. 2017;18(7):452‐465. 28488703 10.1038/nrm.2017.22 PMC5667541

[7] Grabarics M , Lettow M , Kirschbaum C , Greis K , Manz C , Pagel K . Mass spectrometry‐based techniques to elucidate the sugar code. Chem Rev. 2022;122(8):7840‐7908. 34491038 10.1021/acs.chemrev.1c00380 PMC9052437

[8] Chatham JC , Zhang J , Wende AR . Role of O‐linked N‐acetylglucosamine protein modification in cellular (patho)physiology. Physiol Rev. 2021;101(2):427‐493. 32730113 10.1152/physrev.00043.2019 PMC8428922

[9] Torres CR , Hart GW . Topography and polypeptide distribution of terminal N‐acetylglucosamine residues on the surfaces of intact lymphocytes. Evidence for O‐linked GlcNAc. J Biol Chem. 1984;259(5):3308‐3317. 6421821

[10] Holt GD , Hart GW . The subcellular distribution of terminal N‐acetylglucosamine moieties. Localization of a novel protein‐saccharide linkage, O‐linked GlcNAc. J Biol Chem. 1986;261(17):8049‐8057. 3086323

[11] Vocadlo DJ , Hang HC , Kim E‐J , Hanover JA , Bertozzi CR . A chemical approach for identifying O‐GlcNAc‐modified proteins in cells. PNAS. 2003;100(16):9116–9121. 12874386 10.1073/pnas.1632821100 PMC171382

[12] Holt GD , Haltiwanger RS , Torres CR , Hart GW . Erythrocytes contain cytoplasmic glycoproteins. O‐linked GlcNAc on Band 4.1. J Biol Chem. 1987;262(31):14847–14850. 3117790

[13] Snow CM , Senior A , Gerace L . Monoclonal antibodies identify a group of nuclear pore complex glycoproteins. J Cell Biol. 1987;104(5):1143‐1156. 2437126 10.1083/jcb.104.5.1143 PMC2114474

[14] Holt GD , Snow CM , Senior A , Haltiwanger RS , Gerace L , Hart GW . Nuclear pore complex glycoproteins contain cytoplasmically disposed O‐linked N‐acetylglucosamine. J Cell Biol. 1987;104(5):1157‐1164. 3571327 10.1083/jcb.104.5.1157 PMC2114481

[15] Turner JR , Tartakoff AM , Greenspan NS . Cytologic assessment of nuclear and cytoplasmic O‐linked N‐acetylglucosamine distribution by using anti‐streptococcal monoclonal antibodies. Proc Natl Acad Sci USA. 1990;87(15):5608‐5612. 2116002 10.1073/pnas.87.15.5608 PMC54376

[16] Holt GD , Haltiwanger RS , Torres CR , Hart GW . Erythrocytes contain cytoplasmic glycoproteins. O‐linked GlcNAc on Band 4.1. J Biol Chem. 1987;262(31):14847‐14850. 3117790

[17] Haltiwanger RS , Blomberg MA , Hart GW . Glycosylation of nuclear and cytoplasmic proteins. Purification and characterization of a uridine diphospho‐N‐acetylglucosamine:polypeptide beta‐N‐acetylglucosaminyltransferase. J Biol Chem. 1992;267(13):9005‐9013. 1533623

[18] Haltiwanger RS , Holt GD , Hart GW . Enzymatic addition of O‐GlcNAc to nuclear and cytoplasmic proteins. Identification of a uridine diphospho‐N‐acetylglucosamine:peptide beta‐N‐acetylglucosaminyltransferase. J Biol Chem. 1990;265(5):2563‐2568. 2137449

[19] Kreppel LK , Blomberg MA , Hart GW . Dynamic glycosylation of nuclear and cytosolic proteins. Cloning and characterization of a unique O‐GlcNAc transferase with multiple tetratricopeptide repeats. J Biol Chem. 1997;272(14):9308‐9315. 9083067 10.1074/jbc.272.14.9308

[20] Li X , Yue X , Sepulveda H , et al. OGT controls mammalian cell viability by regulating the proteasome/mTOR/mitochondrial axis. Proc Natl Acad Sci USA. 2023;120(3):e2218332120. 36626549 10.1073/pnas.2218332120 PMC9934350

[21] Hart G . Nutrient regulation of signaling and transcription. J Biol Chem. 2019;294(7):2211‐2231. 30626734 10.1074/jbc.AW119.003226 PMC6378989

[22] Hart G , Slawson C , Ramirez‐Correa G , Lagerlof O . Cross talk between O‐GlcNAcylation and phosphorylation: roles in signaling, transcription, and chronic disease. Annu Rev Biochem. 2011;80:825‐858. 21391816 10.1146/annurev-biochem-060608-102511 PMC3294376

[23] Zachara N , Hart G . O‐GlcNAc a sensor of cellular state: the role of nucleocytoplasmic glycosylation in modulating cellular function in response to nutrition and stress. Biochim Biophys Acta. 2004;1673:13‐28. 15238246 10.1016/j.bbagen.2004.03.016

[24] Milewski S . Glucosamine‐6‐phosphate synthase–the multi‐facets enzyme. Biochim Biophys Acta. 2002;1597(2):173‐192. 12044898 10.1016/s0167-4838(02)00318-7

[25] Oki T , Yamazaki K , Kuromitsu J , Okada M , Tanaka I . cDNA cloning and mapping of a novel subtype of glutamine:fructose‐6‐phosphate amidotransferase (GFAT2) in human and mouse. Genomics. 1999;57(2):227‐234. 10198162 10.1006/geno.1999.5785

[26] Chang Q , Su K , Baker J , Yang X , Paterson A , Kudlow J . Phosphorylation of human glutamine:fructose‐6‐phosphate amidotransferase by cAMP‐dependent protein kinase at serine 205 blocks the enzyme activity. J Biol Chem. 2000;275(29):21981‐21987. 10806197 10.1074/jbc.M001049200

[27] Hu Y , Riesland L , Paterson A , Kudlow J . Phosphorylation of mouse glutamine‐fructose‐6‐phosphate amidotransferase 2 (GFAT2) by cAMP‐dependent protein kinase increases the enzyme activity. J Biol Chem. 2004;279(29):29988‐29993. 15133036 10.1074/jbc.M401547200

[28] Li Y , Roux C , Lazereg S , et al. Identification of a novel serine phosphorylation site in human glutamine:fructose‐6‐phosphate amidotransferase isoform 1. Biochemistry. 2007;46(45):13163‐13169. 17941647 10.1021/bi700694c

[29] Eguchi S , Oshiro N , Miyamoto T , et al. AMP‐activated protein kinase phosphorylates glutamine : fructose‐6‐phosphate amidotransferase 1 at Ser243 to modulate its enzymatic activity. Genes Cells. 2009;14(2):179‐189. 19170765 10.1111/j.1365-2443.2008.01260.x

[30] Sayeski P , Wang D , Su K , Han I , Kudlow J . Cloning and partial characterization of the mouse glutamine:fructose‐6‐phosphate amidotransferase (GFAT) gene promoter. Nucleic Acids Res. 1997;25(7):1458‐1466. 9060444 10.1093/nar/25.7.1458 PMC146605

[31] Chaveroux C , Sarcinelli C , Barbet V , et al. Nutrient shortage triggers the hexosamine biosynthetic pathway via the GCN2‐ATF4 signalling pathway. Sci Rep. 2016;6:27278. 27255611 10.1038/srep27278 PMC4891703

[32] Qian Y , Ahmad M , Chen S , et al. Discovery of 1‐arylcarbonyl‐6,7‐dimethoxyisoquinoline derivatives as glutamine fructose‐6‐phosphate amidotransferase (GFAT) inhibitors. Bioorg Med Chem Lett. 2011;21(21):6264‐6269. 21958546 10.1016/j.bmcl.2011.09.009

[33] Vyas B , Silakari O , Bahia M , Singh B . Glutamine: fructose‐6‐phosphate amidotransferase (GFAT): homology modelling and designing of new inhibitors using pharmacophore and docking based hierarchical virtual screening protocol. SAR QSAR Environ Res. 2013;24(9):733‐752. 23767808 10.1080/1062936X.2013.797493

[34] Boehmelt G , Fialka I , Brothers G , et al. Cloning and characterization of the murine glucosamine‐6‐phosphate acetyltransferase EMeg32. Differential expression and intracellular membrane association. J Biol Chem. 2000;275(17):12821‐12832. 10777580 10.1074/jbc.275.17.12821

[35] Mio T , Yabe T , Arisawa M , Yamada‐Okabe H . The eukaryotic UDP‐N‐acetylglucosamine pyrophosphorylases. Gene cloning, protein expression, and catalytic mechanism. J Biol Chem. 1998;273(23):14392‐14397. 9603950 10.1074/jbc.273.23.14392

[36] Gibb A , Lorkiewicz P , Zheng Y , et al. Correction: integration of flux measurements to resolve changes in anabolic and catabolic metabolism in cardiac myocytes. Biochem J. 2017;474(24):4271‐4272. 29242383 10.1042/BCJ20170474_COR PMC5735646

[37] Olson A , Bouchard B , Zhu W , Chatham J , Des Rosiers C . ex vivoFirst characterization of glucose flux through the hexosamine biosynthesis pathway (HBP) in mouse heart. J Biol Chem. 2020;295(7):2018‐2033. 31915250 10.1074/jbc.RA119.010565 PMC7029105

[38] Kreppel L , Blomberg M , Hart G . Dynamic glycosylation of nuclear and cytosolic proteins. Cloning and characterization of a unique O‐GlcNAc transferase with multiple tetratricopeptide repeats. J Biol Chem. 1997;272(14):9308‐9315. 9083067 10.1074/jbc.272.14.9308

[39] Haltiwanger R , Blomberg M , Hart G . Glycosylation of nuclear and cytoplasmic proteins. Purification and characterization of a uridine diphospho‐N‐acetylglucosamine:polypeptide beta‐N‐acetylglucosaminyltransferase. J Biol Chem. 1992;267(13):9005‐9013. 1533623

[40] Lubas W , Frank D , Krause M , Hanover J . O‐Linked GlcNAc transferase is a conserved nucleocytoplasmic protein containing tetratricopeptide repeats. J Biol Chem. 1997;272(14):9316‐9324. 9083068 10.1074/jbc.272.14.9316

[41] Zeytuni N , Zarivach R . Structural and functional discussion of the tetra‐trico‐peptide repeat, a protein interaction module. Structure. 2012;20(3):397‐405. 22404999 10.1016/j.str.2012.01.006

[42] Allan R , Ratajczak T . Versatile TPR domains accommodate different modes of target protein recognition and function. Cell Stress Chaperones. 2011;16(4):353‐367. 21153002 10.1007/s12192-010-0248-0 PMC3118826

[43] Lazarus M , Nam Y , Jiang J , Sliz P , Walker S . Structure of human O‐GlcNAc transferase and its complex with a peptide substrate. Nature. 2011;469(7331):564‐567. 21240259 10.1038/nature09638 PMC3064491

[44] Janetzko J , Walker S . The making of a sweet modification: structure and function of O‐GlcNAc transferase. J Biol Chem. 2014;289(50):34424‐34432. 25336649 10.1074/jbc.R114.604405 PMC4263849

[45] Levine Z , Walker S . The biochemistry of O‐GlcNAc transferase: which functions make it essential in mammalian cells? Annu Rev Biochem. 2016;85:631‐657. 27294441 10.1146/annurev-biochem-060713-035344

[46] Hanover J , Forsythe M , Hennessey P , et al. A Caenorhabditis elegans model of insulin resistance: altered macronutrient storage and dauer formation in an OGT‐1 knockout. Proc Natl Acad Sci USA. 2005;102(32):11266‐11271. 16051707 10.1073/pnas.0408771102 PMC1183534

[47] Gambetta M , Oktaba K , Müller J . Essential role of the glycosyltransferase sxc/Ogt in polycomb repression. Science. 2009;325(5936):93‐96. 19478141 10.1126/science.1169727

[48] Sinclair D , Syrzycka M , Macauley M , et al. Drosophila O‐GlcNAc transferase (OGT) is encoded by the Polycomb group (PcG) gene, super sex combs (sxc). Proc Natl Acad Sci USA. 2009;106(32):13427‐13432. 19666537 10.1073/pnas.0904638106 PMC2726349

[49] Kazemi Z , Chang H , Haserodt S , McKen C , Zachara N . O‐linked beta‐N‐acetylglucosamine (O‐GlcNAc) regulates stress‐induced heat shock protein expression in a GSK‐3beta‐dependent manner. J Biol Chem. 2010;285(50):39096‐39107. 20926391 10.1074/jbc.M110.131102 PMC2998145

[50] Shafi R , Iyer S , Ellies L , et al. The O‐GlcNAc transferase gene resides on the X chromosome and is essential for embryonic stem cell viability and mouse ontogeny. Proc Natl Acad Sci USA. 2000;97(11):5735‐5739. 10801981 10.1073/pnas.100471497 PMC18502

[51] O'Donnell N , Zachara N , Hart G , Marth J . Ogt‐dependent X‐chromosome‐linked protein glycosylation is a requisite modification in somatic cell function and embryo viability. Mol Cell Biol. 2004;24(4):1680‐1690. 14749383 10.1128/MCB.24.4.1680-1690.2004 PMC344186

[52] Ruan H , Dietrich M , Liu Z , et al. O‐GlcNAc transferase enables AgRP neurons to suppress browning of white fat. Cell. 2014;159(2):306‐317. 25303527 10.1016/j.cell.2014.09.010 PMC4509746

[53] Watson L , Long B , DeMartino A , et al. Cardiomyocyte Ogt is essential for postnatal viability. Am J Physiol. 2014;306(1):H142‐H153. 10.1152/ajpheart.00438.2013 PMC3920156 24186210

[54] Agah R , Frenkel P , French B , Michael L , Overbeek P , Schneider M . Gene recombination in postmitotic cells. Targeted expression of Cre recombinase provokes cardiac‐restricted, site‐specific rearrangement in adult ventricular muscle in vivo. J Clin Invest. 1997;100(1):169‐179. 9202069 10.1172/JCI119509 PMC508177

[55] Nagel A , Ball L . O‐GlcNAc transferase and O‐GlcNAcase: achieving target substrate specificity. Amino Acids. 2014;46(10):2305‐2316. 25173736 10.1007/s00726-014-1827-7 PMC4584397

[56] Whelan S , Lane M , Hart G . Regulation of the O‐linked beta‐N‐acetylglucosamine transferase by insulin signaling. J Biol Chem. 2008;283(31):21411‐21417. 18519567 10.1074/jbc.M800677200 PMC2490780

[57] Yang Y , Li X , Luan H , et al. OGT suppresses S6K1‐mediated macrophage inflammation and metabolic disturbance. Proc Natl Acad Sci USA. 2020;117(28):16616‐16625. 32601203 10.1073/pnas.1916121117 PMC7368321

[58] Kaasik K , Kivimäe S , Allen J , et al. Glucose sensor O‐GlcNAcylation coordinates with phosphorylation to regulate circadian clock. Cell Metab. 2013;17(2):291‐302. 23395175 10.1016/j.cmet.2012.12.017 PMC3597447

[59] Bullen J , Balsbaugh J , Chanda D , et al. Cross‐talk between two essential nutrient‐sensitive enzymes: o‐GlcNAc transferase (OGT) and AMP‐activated protein kinase (AMPK). J Biol Chem. 2014;289(15):10592‐10606. 24563466 10.1074/jbc.M113.523068 PMC4036179

[60] Li Z , Li X , Nai S , et al. O‐Checkpoint kinase 1‐induced phosphorylation of linked β–acetylglucosamine transferase regulates the intermediate filament network during cytokinesis. J Biol Chem. 2017;292(48):19548‐19555. 29021254 10.1074/jbc.M117.811646 PMC5712597

[61] Ruan H , Ma Y , Torres S , et al. Calcium‐dependent O‐GlcNAc signaling drives liver autophagy in adaptation to starvation. Genes Dev. 2017;31(16):1655‐1665. 28903979 10.1101/gad.305441.117 PMC5647936

[62] Seo H , Kim H , Kang M , Ryum J , Yi E , Cho J . Identification of the nuclear localisation signal of O‐GlcNAc transferase and its nuclear import regulation. Sci Rep. 2016;6:34614. 27713473 10.1038/srep34614 PMC5054401

[63] Ruan H , Han X , Li M , et al. O‐GlcNAc transferase/host cell factor C1 complex regulates gluconeogenesis by modulating PGC‐1α stability. Cell Metab. 2012;16(2):226‐237. 22883232 10.1016/j.cmet.2012.07.006 PMC3480732

[64] Cheung W , Hart G . AMP‐activated protein kinase and p38 MAPK activate O‐GlcNAcylation of neuronal proteins during glucose deprivation. J Biol Chem. 2008;283(19):13009‐13020. 18353774 10.1074/jbc.M801222200 PMC2435304

[65] Berthier A , Vinod M , Porez G , et al. Combinatorial regulation of hepatic cytoplasmic signaling and nuclear transcriptional events by the OGT/REV‐ERBα complex. Proc Natl Acad Sci USA. 2018;115(47):E11033‐E11042. 30397120 10.1073/pnas.1805397115 PMC6255172

[66] Whisenhunt T , Yang X , Bowe D , Paterson A , Van Tine B , Kudlow J . Disrupting the enzyme complex regulating O‐GlcNAcylation blocks signaling and development. Glycobiology. 2006;16(6):551‐563. 16505006 10.1093/glycob/cwj096

[67] Ferrer C , Lynch T , Sodi V , et al. O‐GlcNAcylation regulates cancer metabolism and survival stress signaling via regulation of the HIF‐1 pathway. Mol Cell. 2014;54(5):820‐831. 24857547 10.1016/j.molcel.2014.04.026 PMC4104413

[68] Sodi V , Khaku S , Krutilina R , et al. mTOR/MYC axis regulates O‐GlcNAc transferase expression and O‐GlcNAcylation in breast cancer. Mol Cancer Res. 2015;13(5):923‐933. 25636967 10.1158/1541-7786.MCR-14-0536 PMC4433402

[69] Ferrer C , Lu T , Bacigalupa Z , Katsetos C , Sinclair D , Reginato M . O‐GlcNAcylation regulates breast cancer metastasis via SIRT1 modulation of FOXM1 pathway. Oncogene. 2017;36(4):559‐569. 27345396 10.1038/onc.2016.228 PMC5192006

[70] Yang X , Ongusaha P , Miles P , et al. Phosphoinositide signalling links O‐GlcNAc transferase to insulin resistance. Nature. 2008;451(7181):964‐969. 18288188 10.1038/nature06668

[71] Joiner C , Levine Z , Aonbangkhen C , Woo C , Walker S . Aspartate residues far from the active site drive O‐GlcNAc transferase substrate selection. J Am Chem Soc. 2019;141(33):12974‐12978. 31373491 10.1021/jacs.9b06061 PMC6849375

[72] Capotosti F , Guernier S , Lammers F , et al. O‐GlcNAc transferase catalyzes site‐specific proteolysis of HCF‐1. Cell. 2011;144(3):376‐388. 21295698 10.1016/j.cell.2010.12.030

[73] Daou S , Mashtalir N , Hammond‐Martel I , et al. Crosstalk between O‐GlcNAcylation and proteolytic cleavage regulates the host cell factor‐1 maturation pathway. Proc Natl Acad Sci USA. 2011;108(7):2747‐27452. 21285374 10.1073/pnas.1013822108 PMC3041071

[74] Lazarus M , Jiang J , Kapuria V , et al. HCF‐1 is cleaved in the active site of O‐GlcNAc transferase. Science. 2013;342(6163):1235‐1239. 24311690 10.1126/science.1243990 PMC3930058

[75] Gross B , Kraybill B , Walker S . Discovery of O‐GlcNAc transferase inhibitors. J Am Chem Soc. 2005;127(42):14588‐14589. 16231908 10.1021/ja0555217

[76] Jiang J , Lazarus M , Pasquina L , Sliz P , Walker S . A neutral diphosphate mimic crosslinks the active site of human O‐GlcNAc transferase. Nat Chem Biol. 2011;8(1):72‐77. 22082911 10.1038/nchembio.711 PMC3241908

[77] Banerjee P , Hart G , Cho J . Chemical approaches to study O‐GlcNAcylation. Chem Soc Rev. 2013;42(10):4345‐4357. 23247267 10.1039/c2cs35412h PMC3641162

[78] Ngoh G , Watson L , Facundo H , Dillmann W , Jones S . Non‐canonical glycosyltransferase modulates post‐hypoxic cardiac myocyte death and mitochondrial permeability transition. J Mol Cell Cardiol. 2008;45(2):313‐325. 18539296 10.1016/j.yjmcc.2008.04.009 PMC2610867

[79] Zafir A , Readnower R , Long B , et al. Protein O‐GlcNAcylation is a novel cytoprotective signal in cardiac stem cells. Stem Cells. 2013;31(4):765‐775. 23335157 10.1002/stem.1325 PMC3606688

[80] Gloster T , Zandberg W , Heinonen J , Shen D , Deng L , Vocadlo D . Hijacking a biosynthetic pathway yields a glycosyltransferase inhibitor within cells. Nat Chem Biol. 2011;7(3):174‐181. 21258330 10.1038/nchembio.520 PMC3202988

[81] Dong D , Hart G . Purification and characterization of an O‐GlcNAc selective N‐acetyl‐beta‐D‐glucosaminidase from rat spleen cytosol. J Biol Chem. 1994;269(30):19321‐19330. 8034696

[82] Toleman C , Paterson A , Kudlow J . Location and characterization of the O‐GlcNAcase active site. Biochim Biophys Acta. 2006;1760(5):829‐839. 16517082 10.1016/j.bbagen.2006.01.017

[83] Clark R , McDonough P , Swanson E , et al. Diabetes and the accompanying hyperglycemia impairs cardiomyocyte calcium cycling through increased nuclear O‐GlcNAcylation. J Biol Chem. 2003;278(45):44230‐44237. 12941958 10.1074/jbc.M303810200

[84] Heckel D , Comtesse N , Brass N , Blin N , Zang K , Meese E . Novel immunogenic antigen homologous to hyaluronidase in meningioma. Hum Mol Genet. 1998;7(12):1859‐1872. 9811929 10.1093/hmg/7.12.1859

[85] Joiner C , Li H , Jiang J , Walker S . Structural characterization of the O‐GlcNAc cycling enzymes: insights into substrate recognition and catalytic mechanisms. Curr Opin Struct Biol. 2019;56:97‐106. 30708324 10.1016/j.sbi.2018.12.003 PMC6656603

[86] Kohler J . Carb cutting works better with a partner. Nat Struct Mol Biol. 2017;24(5):433‐435. 28471427 10.1038/nsmb.3405

[87] Rao F , Schüttelkopf A , Dorfmueller H , Ferenbach A , Navratilova I , van Aalten D . Structure of a bacterial putative acetyltransferase defines the fold of the human O‐GlcNAcase C‐terminal domain. Open Biol. 2013;3(10):130021. 24088714 10.1098/rsob.130021 PMC3814719

[88] Bond M , Hanover J . A little sugar goes a long way: the cell biology of O‐GlcNAc. J Cell Biol. 2015;208(7):869‐880. 25825515 10.1083/jcb.201501101 PMC4384737

[89] Roth C , Chan S , Offen W , et al. Structural and functional insight into human O‐GlcNAcase. Nat Chem Biol. 2017;13(6):610‐612. 28346405 10.1038/nchembio.2358 PMC5438047

[90] Elsen N , Patel S , Ford R , et al. Insights into activity and inhibition from the crystal structure of human O‐GlcNAcase. Nat Chem Biol. 2017;13(6):613‐615. 28346407 10.1038/nchembio.2357

[91] Li B , Li H , Lu L , Jiang J . Structures of human O‐GlcNAcase and its complexes reveal a new substrate recognition mode. Nat Struct Mol Biol. 2017;24(4):362‐369. 28319083 10.1038/nsmb.3390 PMC8171356

[92] Shen D , Gloster T , Yuzwa S , Vocadlo D . Insights into O‐linked N‐acetylglucosamine ([0‐9]O‐GlcNAc) processing and dynamics through kinetic analysis of O‐GlcNAc transferase and O‐GlcNAcase activity on protein substrates. J Biol Chem. 2012;287(19):15395‐15408. 22311971 10.1074/jbc.M111.310664 PMC3346082

[93] Wells L , Gao Y , Mahoney J , et al. Dynamic O‐glycosylation of nuclear and cytosolic proteins: further characterization of the nucleocytoplasmic beta‐N‐acetylglucosaminidase, O‐GlcNAcase. J Biol Chem. 2002;277(3):1755‐1761. 11788610 10.1074/jbc.m109656200

[94] Dentin R , Hedrick S , Xie J , Yates J , Montminy M . Hepatic glucose sensing via the CREB coactivator CRTC2. Science. 2008;319(5868):1402‐1405. 18323454 10.1126/science.1151363

[95] Housley M , Rodgers J , Udeshi N , et al. O‐GlcNAc regulates FoxO activation in response to glucose. J Biol Chem. 2008;283(24):16283‐16292. 18420577 10.1074/jbc.M802240200 PMC2423255

[96] Jackson S , Tjian R . O‐glycosylation of eukaryotic transcription factors: implications for mechanisms of transcriptional regulation. Cell. 1988;55(1):125‐133. 3139301 10.1016/0092-8674(88)90015-3

[97] Han I , Kudlow J . Reduced O glycosylation of Sp1 is associated with increased proteasome susceptibility. Mol Cell Biol. 1997;17(5):2550‐2558. 9111324 10.1128/mcb.17.5.2550 PMC232104

[98] Zhang F , Su K , Yang X , Bowe D , Paterson A , Kudlow J . O‐GlcNAc modification is an endogenous inhibitor of the proteasome. Cell. 2003;115(6):715‐725. 14675536 10.1016/s0092-8674(03)00974-7

[99] Lim K , Chang H . O‐GlcNAc inhibits interaction between Sp1 and Elf‐1 transcription factors. Biochem Biophys Res Commun. 2009;380(3):569‐574. 19285002 10.1016/j.bbrc.2009.01.121

[100] Lim K , Chang H . O‐GlcNAcylation of Sp1 interrupts Sp1 interaction with NF‐Y. Biochem Biophys Res Commun. 2009;382(3):593‐597. 19302979 10.1016/j.bbrc.2009.03.075

[101] Ha C , Lim K . O‐GlcNAc modification of Sp3 and Sp4 transcription factors negatively regulates their transcriptional activities. Biochem Biophys Res Commun. 2015;467(2):341‐347. 26431879 10.1016/j.bbrc.2015.09.155

[102] Ramakrishnan P , Clark P , Mason D , Peters E , Hsieh‐Wilson L , Baltimore D . Activation of the transcriptional function of the NF‐κB protein c‐Rel by O‐GlcNAc glycosylation. Sci Signal. 2013;6(290):ra75. 23982206 10.1126/scisignal.2004097 PMC4066889

[103] Kelly W , Dahmus M , Hart G . RNA polymerase II is a glycoprotein. Modification of the COOH‐terminal domain by O‐GlcNAc. J Biol Chem. 1993;268(14):10416‐10424. 8486697

[104] Li X , Molina H , Huang H , et al. O‐linked N‐acetylglucosamine modification on CCAAT enhancer‐binding protein beta: role during adipocyte differentiation. J Biol Chem. 2009;284(29):19248‐19254. 19478079 10.1074/jbc.M109.005678 PMC2740549

[105] Qian K , Wang S , Fu M , et al. Transcriptional regulation of O‐GlcNAc homeostasis is disrupted in pancreatic cancer. J Biol Chem. 2018;293(36):13989‐14000. 30037904 10.1074/jbc.RA118.004709 PMC6130940

[106] Zhang Z , Huang Z , Awad M , et al. O‐GlcNAc glycosylation orchestrates fate decision and niche function of bone marrow stromal progenitors. Elife. 2023;12:e85464. 36861967 10.7554/eLife.85464 PMC10032655

[107] Kamemura K , Hayes BK , Comer FI , Hart GW . Dynamic interplay between O‐glycosylation and O‐phosphorylation of nucleocytoplasmic proteins: alternative glycosylation/phosphorylation of THR‐58, a known mutational hot spot of c‐Myc in lymphomas, is regulated by mitogens. J Biol Chem. 2002;277(21):19229‐19235. 11904304 10.1074/jbc.M201729200

[108] Lee DH , Kwon NE , Lee W‐J , et al. Increased O‐GlcNAcylation of c‐Myc promotes pre‐B cell proliferation. Cells. 2020;9(1):158. 31936366 10.3390/cells9010158 PMC7016991

[109] Zhang J , Yang P , Liu D , et al. Inhibiting Hyper‐O‐GlcNAcylation of c‐Myc accelerate diabetic wound healing by alleviating keratinocyte dysfunction. Burns Trauma. 2021;9:tkab031. 34646892 10.1093/burnst/tkab031 PMC8499626

[110] Itkonen HM , Minner S , Guldvik IJ , et al. O‐GlcNAc transferase integrates metabolic pathways to regulate the stability of c‐MYC in human prostate cancer cells. Cancer Res. 2013;73(16):5277‐5287. 23720054 10.1158/0008-5472.CAN-13-0549

[111] Chou TY , Dang CV , Hart GW . Glycosylation of the c‐Myc transactivation domain. Proc Natl Acad Sci USA. 1995;92(10):4417‐4421. 7753821 10.1073/pnas.92.10.4417 PMC41955

[112] Chou TY , Hart GW , Dang CV . c‐Myc is glycosylated at threonine 58, a known phosphorylation site and a mutational hot spot in lymphomas. J Biol Chem. 1995;270(32):18961‐18965. 7642555 10.1074/jbc.270.32.18961

[113] Rexach JE , Clark PM , Mason DE , Neve RL , Peters EC , Hsieh‐Wilson LC . Dynamic O‐GlcNAc modification regulates CREB‐mediated gene expression and memory formation. Nat Chem Biol. 2012;8(3):253‐261. 22267118 10.1038/nchembio.770 PMC3288555

[114] Groussaud D , Khair M , Tollenaere AI , et al. Hijacking of the O‐GlcNAcZYME complex by the HTLV‐1 Tax oncoprotein facilitates viral transcription. PLoS Path. 2017;13(7):e1006518. 10.1371/journal.ppat.1006518 PMC5542696 28742148

[115] Gao Y , Liu J , Bai Z , et al. Iron down‐regulates leptin by suppressing protein O‐GlcNAc modification in adipocytes, resulting in decreased levels of O‐glycosylated CREB. J Biol Chem. 2019;294(14):5487‐5495. 30709903 10.1074/jbc.RA118.005183 PMC6462527

[116] Cheng X , Hart GW . Glycosylation of the murine estrogen receptor‐alpha. J Steroid Biochem Mol Biol. 2000;75(2‐3):147‐158. 11226831 10.1016/s0960-0760(00)00167-9

[117] Misra J , Kim D‐K , Jung YS , et al. O‐GlcNAcylation of orphan nuclear receptor estrogen‐related receptor γ promotes hepatic gluconeogenesis. Diabetes. 2016;65(10):2835‐2848. 27335230 10.2337/db15-1523

[118] Chen P‐H , Smith TJ , Wu J , et al. Glycosylation of KEAP1 links nutrient sensing to redox stress signaling. EMBO J. 2017;36(15):2233‐2250. 28663241 10.15252/embj.201696113 PMC5538768

[119] Constable S , Lim J‐M , Vaidyanathan K , Wells L . O‐GlcNAc transferase regulates transcriptional activity of human Oct4. Glycobiology. 2017;27(10):927‐937. 28922739 10.1093/glycob/cwx055 PMC6410957

[120] Jang H , Kim TW , Yoon S , et al. O‐GlcNAc regulates pluripotency and reprogramming by directly acting on core components of the pluripotency network. Cell Stem Cell. 2012;11(1):62‐74. 22608532 10.1016/j.stem.2012.03.001

[121] Gurel Z , Sheibani N . O‐Linked β‐N‐acetylglucosamine (O‐GlcNAc) modification: a new pathway to decode pathogenesis of diabetic retinopathy. Clin Sci. 2018;132(2):185‐198. 10.1042/CS20171454 PMC6016849 29352075

[122] Yang WH , Kim JE , Nam HW , et al. Modification of p53 with O‐linked N‐acetylglucosamine regulates p53 activity and stability. Nat Cell Biol. 2006;8(10):1074‐1083. 16964247 10.1038/ncb1470

[123] Gonzalez‐Rellan MJ , Fondevila MF , Fernandez U , et al. O‐GlcNAcylated p53 in the liver modulates hepatic glucose production. Nat Commun. 2021;12(1):5068. 34417460 10.1038/s41467-021-25390-0 PMC8379189

[124] Shtraizent N , DeRossi C , Nayar S , et al. MPI depletion enhances O‐GlcNAcylation of p53 and suppresses the Warburg effect. Elife. 2017;6:e22477. 28644127 10.7554/eLife.22477 PMC5495572

[125] Ruan H‐B , Han X , Li M‐D , et al. O‐GlcNAc transferase/host cell factor C1 complex regulates gluconeogenesis by modulating PGC‐1α stability. Cell Metab. 2012;16(2):226‐237. 22883232 10.1016/j.cmet.2012.07.006 PMC3480732

[126] Ji S , Park SY , Roth J , Kim HS , Cho JW . O‐GlcNAc modification of PPARγ reduces its transcriptional activity. Biochem Biophys Res Commun. 2012;417(4):1158‐1163. 22226965 10.1016/j.bbrc.2011.12.086

[127] Han C , Gu Y , Shan H , et al. O‐GlcNAcylation of SIRT1 enhances its deacetylase activity and promotes cytoprotection under stress. Nat Commun. 2017;8(1):1491. 29133780 10.1038/s41467-017-01654-6 PMC5684413

[128] Chattopadhyay T , Maniyadath B , Bagul HP , et al. Spatiotemporal gating of SIRT1 functions by O‐GlcNAcylation is essential for liver metabolic switching and prevents hyperglycemia. Proc Natl Acad Sci USA. 2020;117(12):6890‐6900. 32152092 10.1073/pnas.1909943117 PMC7104039

[129] Cao Y , Zhang M , Li Y , et al. O‐GlcNAcylation of SIRT1 protects against cold stress‐induced skeletal muscle damage via amelioration of mitochondrial homeostasis. Int J Mol Sci. 2022;23(23):14520. 36498847 10.3390/ijms232314520 PMC9737900

[130] Erickson J , Pereira L , Wang L , et al. Diabetic hyperglycaemia activates CaMKII and arrhythmias by O‐linked glycosylation. Nature. 2013;502(7471):372‐376. 24077098 10.1038/nature12537 PMC3801227

[131] Yokoe S , Asahi M , Takeda T , et al. Inhibition of phospholamban phosphorylation by O‐GlcNAcylation: implications for diabetic cardiomyopathy. Glycobiology. 2010;20(10):1217‐1226. 20484118 10.1093/glycob/cwq071

[132] Song M , Kim H , Park J , et al. o‐GlcNAc transferase is activated by CaMKIV‐dependent phosphorylation under potassium chloride‐induced depolarization in NG‐108‐15 cells. Cell Signal. 2008;20(1):94‐104. 18029144 10.1016/j.cellsig.2007.09.002

[133] Liu Y , Ao X , Jia Y , Li X , Wang Y , Wang J . The FOXO family of transcription factors: key molecular players in gastric cancer. J Mol Med. 2022;100(7):997‐1015. 35680690 10.1007/s00109-022-02219-x

[134] Liu Y , Li X , Zhou X , Wang J , Ao X . FADD as a key molecular player in cancer progression. Mol Med. 2022;28(1):132. 36348274 10.1186/s10020-022-00560-y PMC9644706

[135] Yu F , Zhang Q , Liu H , et al. Dynamic O‐GlcNAcylation coordinates ferritinophagy and mitophagy to activate ferroptosis. Cell Discov. 2022;8(1):40. 35504898 10.1038/s41421-022-00390-6 PMC9065108

[136] Wang X , Liu M , Chu Y , et al. O‐GlcNAcylation of ZEB1 facilitated mesenchymal pancreatic cancer cell ferroptosis. Int J Biol Sci. 2022;18(10):4135‐4150. 35844792 10.7150/ijbs.71520 PMC9274488

[137] Zhu G , Murshed A , Li H , et al. O‐GlcNAcylation enhances sensitivity to RSL3‐induced ferroptosis via the YAP/TFRC pathway in liver cancer. Cell Death Discov. 2021;7(1):83. 33863873 10.1038/s41420-021-00468-2 PMC8052337

[138] Chen Y , Zhu G , Liu Y , et al. O‐GlcNAcylated c‐Jun antagonizes ferroptosis via inhibiting GSH synthesis in liver cancer. Cell Signal. 2019;63:109384. 31394193 10.1016/j.cellsig.2019.109384

[139] Pyo K , Kim C , Lee M , Kim J , Kim K , Baek S . ULK1 O‐GlcNAcylation is crucial for activating VPS34 via ATG14L during autophagy initiation. Cell Rep. 2018;25(10):2878‐2890.e4. 30517873 10.1016/j.celrep.2018.11.042

[140] Jin L , Yuan F , Dai G , et al. Blockage of O‐linked GlcNAcylation induces AMPK‐dependent autophagy in bladder cancer cells. Cell Mol Biol Lett. 2020;25:17. 32174982 10.1186/s11658-020-00208-x PMC7063793

[141] Yu H , Wen L , Mu Y . O‐GlcNAcylation is essential for autophagy in cardiomyocytes. Oxid Med Cell Longev. 2020;2020:5602396. 32850000 10.1155/2020/5602396 PMC7439163

[142] Butkinaree C , Park K , Hart G . O‐linked beta‐N‐acetylglucosamine (O‐GlcNAc): extensive crosstalk with phosphorylation to regulate signaling and transcription in response to nutrients and stress. Biochim Biophys Acta. 2010;1800(2):96‐106. 19647786 10.1016/j.bbagen.2009.07.018 PMC2815129

[143] Pang K , Wang W , Qin J‐X , et al. Role of protein phosphorylation in cell signaling, disease, and the intervention therapy. MedComm. 2022;3(4):e175. 36349142 10.1002/mco2.175 PMC9632491

[144] Bilbrough T , Piemontese E , Seitz O . Dissecting the role of protein phosphorylation: a chemical biology toolbox. Chem Soc Rev. 2022;51(13):5691‐5730. 35726784 10.1039/d1cs00991e

[145] Shindo S , Kakizaki S , Sakaki T , et al. Phosphorylation of nuclear receptors: novelty and therapeutic implications. Pharmacol Ther. 2023;248:108477. 37330113 10.1016/j.pharmthera.2023.108477

[146] Wells L , Vosseller K , Hart G . Glycosylation of nucleocytoplasmic proteins: signal transduction and O‐GlcNAc. Science. 2001;291(5512):2376‐2378. 11269319 10.1126/science.1058714

[147] Chou T , Hart G , Dang C . c‐Myc is glycosylated at threonine 58, a known phosphorylation site and a mutational hot spot in lymphomas. J Biol Chem. 1995;270(32):18961‐18965. 7642555 10.1074/jbc.270.32.18961

[148] Du X , Edelstein D , Dimmeler S , Ju Q , Sui C , Brownlee M . Hyperglycemia inhibits endothelial nitric oxide synthase activity by posttranslational modification at the Akt site. J Clin Invest. 2001;108(9):1341‐1348. 11696579 10.1172/JCI11235 PMC209429

[149] Kronlage M , Dewenter M , Grosso J , et al. O‐GlcNAcylation of histone deacetylase 4 protects the diabetic heart from failure. Circulation. 2019;140(7):580‐594. 31195810 10.1161/CIRCULATIONAHA.117.031942

[150] Dias W , Cheung W , Wang Z , Hart G . Regulation of calcium/calmodulin‐dependent kinase IV by O‐GlcNAc modification. J Biol Chem. 2009;284(32):21327‐21337. 19506079 10.1074/jbc.M109.007310 PMC2755857

[151] Gélinas R , Dontaine J , Horman S , Beauloye C , Bultot L , Bertrand L . AMP‐activated protein kinase and O‐GlcNAcylation, two partners tightly connected to regulate key cellular processes. Front Endocrinol. 2018;9:519. 10.3389/fendo.2018.00519 PMC6146136 30271380

[152] Zibrova D , Vandermoere F , Göransson O , et al. GFAT1 phosphorylation by AMPK promotes VEGF‐induced angiogenesis. Biochem J. 2017;474(6):983‐1001. 28008135 10.1042/BCJ20160980

[153] Gélinas R , Mailleux F , Dontaine J , et al. AMPK activation counteracts cardiac hypertrophy by reducing O‐GlcNAcylation. Nat Commun. 2018;9(1):374. 29371602 10.1038/s41467-017-02795-4 PMC5785516

[154] Li M , Ruan H , Hughes M , et al. O‐GlcNAc signaling entrains the circadian clock by inhibiting BMAL1/CLOCK ubiquitination. Cell Metab. 2013;17(2):303‐310. 23395176 10.1016/j.cmet.2012.12.015 PMC3647362

[155] Ruan H , Nie Y , Yang X . Regulation of protein degradation by O‐GlcNAcylation: crosstalk with ubiquitination. Mol Cell Proteomics 2013;12(12):3489‐3497. 23824911 10.1074/mcp.R113.029751 PMC3861702

[156] Hunter T . The age of crosstalk: phosphorylation, ubiquitination, and beyond. Mol Cell. 2007;28(5):730‐738. 18082598 10.1016/j.molcel.2007.11.019

[157] Vousden K . p53: death star. Cell. 2000;103(5):691‐694. 11114324 10.1016/s0092-8674(00)00171-9

[158] Mariller C , Hardivillé S , Hoedt E , Benaïssa M , Mazurier J , Pierce A . Proteomic approach to the identification of novel delta‐lactoferrin target genes: characterization of DcpS, an mRNA scavenger decapping enzyme. Biochimie. 2009;91(1):109‐122. 18725266 10.1016/j.biochi.2008.07.009

[159] Yang W , Kim J , Nam H , et al. Modification of p53 with O‐linked N‐acetylglucosamine regulates p53 activity and stability. Nat Cell Biol. 2006;8(10):1074‐1083. 16964247 10.1038/ncb1470

[160] Hardivillé S , Hoedt E , Mariller C , Benaïssa M , Pierce A . O‐GlcNAcylation/phosphorylation cycling at Ser10 controls both transcriptional activity and stability of delta‐lactoferrin. J Biol Chem. 2010;285(25):19205‐19218. 20404350 10.1074/jbc.M109.080572 PMC2885199

[161] St‐Denis N , Litchfield D . Protein kinase CK2 in health and disease: from birth to death: the role of protein kinase CK2 in the regulation of cell proliferation and survival. Cell Mol Life Sci. 2009;66:1817‐1829. 19387552 10.1007/s00018-009-9150-2 PMC11115660

[162] Tarrant M , Rho H , Xie Z , et al. Regulation of CK2 by phosphorylation and O‐GlcNAcylation revealed by semisynthesis. Nat Chem Biol. 2012;8(3):262‐269. 22267120 10.1038/nchembio.771 PMC3288285

[163] Dentin R , Liu Y , Koo S , et al. Insulin modulates gluconeogenesis by inhibition of the coactivator TORC2. Nature. 2007;449(7160):366‐369. 17805301 10.1038/nature06128

[164] Shrikhande G , Scali S , Silva C , et al. O‐glycosylation regulates ubiquitination and degradation of the anti‐inflammatory protein A20 to accelerate atherosclerosis in diabetic ApoE‐null mice. PLoS One. 2010;5(12):e14240. 21151899 10.1371/journal.pone.0014240 PMC2997780

[165] Srikanth B , Vaidya M , Kalraiya R . O‐GlcNAcylation determines the solubility, filament organization, and stability of keratins 8 and 18. J Biol Chem. 2010;285(44):34062‐34071. 20729549 10.1074/jbc.M109.098996 PMC2962505

[166] Chen P , Smith T , Wu J , et al. Glycosylation of KEAP1 links nutrient sensing to redox stress signaling. EMBO J. 2017;36(15):2233‐2250. 28663241 10.15252/embj.201696113 PMC5538768

[167] Huang H , Wu Q , Guo X , et al. O‐GlcNAcylation promotes the migratory ability of hepatocellular carcinoma cells via regulating FOXA2 stability and transcriptional activity. J Cell Physiol. 2021;236(11):7491‐7503. 33843053 10.1002/jcp.30385

[168] Roth G , Mensah G , Johnson C , et al. Global burden of cardiovascular diseases and risk factors, 1990–2019: update from the GBD 2019 study. J Am Coll Cardiol. 2020;76(25):2982‐3021. 33309175 10.1016/j.jacc.2020.11.010 PMC7755038

[169] Wang H , Wang Y , Zhou Y , et al. Protein O‐GlcNAcylation in cardiovascular diseases. Acta Pharmacol Sin. 2023;44(1):8‐18. 35817809 10.1038/s41401-022-00934-2 PMC9813366

[170] Ao X , Ding W , Li X , et al. Non‐coding RNAs regulating mitochondrial function in cardiovascular diseases. J Mol Med. 2023;101(5):501‐526. 37014377 10.1007/s00109-023-02305-8

[171] Carlisi M , Mancuso S , Lo Presti R , Siragusa S , Caimi G . High output heart failure in multiple myeloma: pathogenetic considerations. Cancers. 2022;14(3):610. 35158878 10.3390/cancers14030610 PMC8833382

[172] Lunde I , Aronsen J , Kvaløy H , et al. Cardiac O‐GlcNAc signaling is increased in hypertrophy and heart failure. Physiol Genomics. 2012;44(2):162‐172. 22128088 10.1152/physiolgenomics.00016.2011

[173] Umapathi P , Mesubi O , Banerjee P , et al. OExcessive ‐GlcNAcylation causes heart failure and sudden death. Circulation. 2021;143(17):1687‐1703. 33593071 10.1161/CIRCULATIONAHA.120.051911 PMC8085112

[174] Arany Z , He H , Lin J , et al. Transcriptional coactivator PGC‐1 alpha controls the energy state and contractile function of cardiac muscle. Cell Metab. 2005;1(4):259‐271. 16054070 10.1016/j.cmet.2005.03.002

[175] Brainard R , Facundo H . Cardiac hypertrophy drives PGC‐1α suppression associated with enhanced O‐glycosylation. Biochim Biophys Acta Mol Bas Dis. 2021;1867(5):166080. 10.1016/j.bbadis.2021.166080 33486096

[176] Li Z , Xu J , Song Y , et al. PRMT5 prevents dilated cardiomyopathy via suppression of protein O‐GlcNAcylation. Circ Res. 2021;129(9):857‐871. 34503365 10.1161/CIRCRESAHA.121.319456

[177] Jones S , Zachara N , Ngoh G , et al. Cardioprotection by N‐acetylglucosamine linkage to cellular proteins. Circulation. 2008;117(9):1172‐1182. 18285568 10.1161/CIRCULATIONAHA.107.730515

[178] Champattanachai V , Marchase R , Chatham J . Glucosamine protects neonatal cardiomyocytes from ischemia‐reperfusion injury via increased protein O‐GlcNAc and increased mitochondrial Bcl‐2. Am J physiol. 2008;294(6):C1509‐C1520. 10.1152/ajpcell.00456.2007 PMC2800950 18367586

[179] Laczy B , Marsh S , Brocks C , Wittmann I , Chatham J . Inhibition of O‐GlcNAcase in perfused rat hearts by NAG‐thiazolines at the time of reperfusion is cardioprotective in an O‐GlcNAc‐dependent manner. Am J Physiol. 2010;299(5):H1715‐H1727. 10.1152/ajpheart.00337.2010 PMC2993218 20833964

[180] Ngoh G , Facundo H , Hamid T , Dillmann W , Zachara N , Jones S . Unique hexosaminidase reduces metabolic survival signal and sensitizes cardiac myocytes to hypoxia/reoxygenation injury. Circ Res. 2009;104(1):41‐49. 19023128 10.1161/CIRCRESAHA.108.189431 PMC2712829

[181] Si R , Zhang Q , Tsuji‐Hosokawa A , et al. Overexpression of p53 due to excess protein O‐GlcNAcylation is associated with coronary microvascular disease in type 2 diabetes. Cardiovasc Res. 2020;116(6):1186‐1198. 31504245 10.1093/cvr/cvz216 PMC7177511

[182] Lu S , Liao Z , Lu X , et al. OHyperglycemia acutely increases cytosolic reactive oxygen species via ‐linked GlcNAcylation and CaMKII activation in mouse ventricular myocytes. Circ Res. 2020;126(10):e80‐e96. 32134364 10.1161/CIRCRESAHA.119.316288 PMC7210078

[183] Hegyi B , Fasoli A , Ko C , et al. CaMKII Serine 280 O‐GlcNAcylation links diabetic hyperglycemia to proarrhythmia. Circ Res. 2021;129(1):98‐113. 33926209 10.1161/CIRCRESAHA.120.318402 PMC8221539

[184] Cividini F , Scott B , Dai A , et al. O‐GlcNAcylation of 8‐oxoguanine DNA glycosylase (Ogg1) impairs oxidative mitochondrial DNA lesion repair in diabetic hearts. J Biol Chem. 2016;291(51):26515‐26528. 27816939 10.1074/jbc.M116.754481 PMC5159511

[185] Prakoso D , Lim S , Erickson J , et al. Fine‐tuning the cardiac O‐GlcNAcylation regulatory enzymes governs the functional and structural phenotype of the diabetic heart. Cardiovasc Res. 2022;118(1):212‐225. 33576380 10.1093/cvr/cvab043

[186] Marsh S , Powell P , Dell'italia L , Chatham J . Cardiac O‐GlcNAcylation blunts autophagic signaling in the diabetic heart. Life Sci. 2013;92(11):648‐656. 22728715 10.1016/j.lfs.2012.06.011 PMC3477499

[187] Laczy B , Fülöp N , Onay‐Besikci A , Des Rosiers C , Chatham J . Acute regulation of cardiac metabolism by the hexosamine biosynthesis pathway and protein O‐GlcNAcylation. PLoS One. 2011;6(4):e18417. 21494549 10.1371/journal.pone.0018417 PMC3073970

[188] Lauzier B , Merlen C , Vaillant F , et al. Post‐translational modifications, a key process in CD36 function: lessons from the spontaneously hypertensive rat heart. J Mol Cell Cardiol. 2011;51(1):99‐108. 21510957 10.1016/j.yjmcc.2011.04.001

[189] Mu Y , Yu H , Wu T , Zhang J , Evans SM , Chen J . O‐linked β‐N‐acetylglucosamine transferase plays an essential role in heart development through regulating angiopoietin‐1. PLoS Genet. 2020;16(4):e1008730. 32251422 10.1371/journal.pgen.1008730 PMC7182263

[190] Shi Q , Shen Q , Liu Y , et al. Increased glucose metabolism in TAMs fuels O‐GlcNAcylation of lysosomal Cathepsin B to promote cancer metastasis and chemoresistance. Cancer Cell. 2022;40(10):1207‐1222. 36084651 10.1016/j.ccell.2022.08.012

[191] Yang Y , Yan Y , Yin J , et al. O‐GlcNAcylation of YTHDF2 promotes HBV‐related hepatocellular carcinoma progression in an N6‐methyladenosine‐dependent manner. Signal Transduct Target Ther. 2023;8(1):63. 36765030 10.1038/s41392-023-01316-8 PMC9918532

[192] Yang Y , Li X , Luan HH , et al. OGT suppresses S6K1‐mediated macrophage inflammation and metabolic disturbance. Proc Natl Acad Sci USA. 2020;117(28):16616‐16625. 32601203 10.1073/pnas.1916121117 PMC7368321

[193] Liu B , Salgado OC , Singh S , et al. The lineage stability and suppressive program of regulatory T cells require protein O‐GlcNAcylation. Nat Commun. 2019;10(1):354. 30664665 10.1038/s41467-019-08300-3 PMC6341091

[194] Lu S , Yin X , Wang J , et al. SIRT1 regulates O‐GlcNAcylation of tau through OGT. Aging. 2020;12(8):7042‐7055. 32310828 10.18632/aging.103062 PMC7202539

[195] Ou W , Liang Y , Qin Y , et al. Hypoxic acclimation improves cardiac redox homeostasis and protects heart against ischemia‐reperfusion injury through upregulation of O‐GlcNAcylation. Redox Biol. 2021;43:101994. 33964586 10.1016/j.redox.2021.101994 PMC8121980

[196] Wang D , Hu X , Lee S , et al. Diabetes exacerbates myocardial ischemia/reperfusion injury by down‐regulation of MicroRNA and up‐regulation of O‐GlcNAcylation. JACC Basic Transl Sci. 2018;3(3):350‐362. 30062222 10.1016/j.jacbts.2018.01.005 PMC6058960

[197] Champattanachai V , Marchase R , Chatham J . Glucosamine protects neonatal cardiomyocytes from ischemia‐reperfusion injury via increased protein‐associated O‐GlcNAc. Ame J Physiol Cell Physiol. 2007;292(1):C178‐C187. 10.1152/ajpcell.00162.2006 16899550

[198] Chun W , Nah D , Bae J , Chung J , Lee H , Moon I . Glucose‐insulin‐potassium solution protects ventricular myocytes of neonatal rat in an in vitro coverslip ischemia/reperfusion model. Korean Circ J. 2015;45(3):234‐241. 26023312 10.4070/kcj.2015.45.3.234 PMC4446818

[199] Jin L , Gao F , Jiang T , et al. Hyper‐O‐GlcNAcylation impairs insulin response against reperfusion‐induced myocardial injury and arrhythmias in obesity. Biochem Biophys Res Commun. 2021;558:126‐133. 33915326 10.1016/j.bbrc.2021.04.066

[200] Akimoto Y , Kreppel L , Hirano H , Hart G . Hyperglycemia and the O‐GlcNAc transferase in rat aortic smooth muscle cells: elevated expression and altered patterns of O‐GlcNAcylation. Arch Biochem Biophys. 2001;389(2):166‐175. 11339805 10.1006/abbi.2001.2331

[201] Rossier B , Bochud M , Devuyst O . The hypertension pandemic: an evolutionary perspective. Physiology. 2017;32(2):112‐125. 28202622 10.1152/physiol.00026.2016

[202] Dos Passos Junior R , Bomfim G , Giachini F , Tostes R , Lima V . NO‐Linked β–acetylglucosamine modification: linking hypertension and the immune system. Front Immunol. 2022;13:852115. 35371030 10.3389/fimmu.2022.852115 PMC8967968

[203] Ng Y , Okolo C , Erickson J , Baldi J , Jones P . Protein O‐GlcNAcylation in the heart. Acta Physiol (Oxf). 2021;233(1):e13696. 34057811 10.1111/apha.13696

[204] Fülöp N , Mason M , Dutta K , et al. Impact of Type 2 diabetes and aging on cardiomyocyte function and O‐linked N‐acetylglucosamine levels in the heart. Am J Physiol Cell Physiol. 2007;292(4):C1370‐C1378. 17135297 10.1152/ajpcell.00422.2006

[205] Silva‐Aguiar R , Bezerra N , Lucena M , et al. O‐GlcNAcylation reduces proximal tubule protein reabsorption and promotes proteinuria in spontaneously hypertensive rats. J Biol Chem. 2018;293(33):12749‐12758. 29954945 10.1074/jbc.RA118.001746 PMC6102134

[206] Lima V , Giachini F , Choi H , et al. Impaired vasodilator activity in deoxycorticosterone acetate‐salt hypertension is associated with increased protein O‐GlcNAcylation. Hypertension. 2009;53(2):166‐174. 19139380 10.1161/HYPERTENSIONAHA.108.116798 PMC2712827

[207] Liu Y , Ding W , Wang J , Ao X , Xue J . Non‐coding RNA‐mediated modulation of ferroptosis in cardiovascular diseases. Biomed Pharmacother. 2023;164:114993. 37302320 10.1016/j.biopha.2023.114993

[208] Dupas T , Denis M , Dontaine J , et al. Protein O‐GlcNAcylation levels are regulated independently of dietary intake in a tissue and time‐specific manner during rat postnatal development. Acta Physiol. 2021;231(3):e13566. 10.1111/apha.13566 PMC7988603 33022862

[209] Liu Y , Wang Y , Li X , Jia Y , Wang J , Ao X . FOXO3a in cancer drug resistance. Cancer Lett. 2022;540:215724. 35545128 10.1016/j.canlet.2022.215724

[210] Zhou X , Ao X , Jia Z , et al. Non‐coding RNA in cancer drug resistance: underlying mechanisms and clinical applications. Front Oncol. 2022;12:951864. 36059609 10.3389/fonc.2022.951864 PMC9428469

[211] Yan W , Cao M , Ruan X , et al. Cancer‐cell‐secreted miR‐122 suppresses O‐GlcNAcylation to promote skeletal muscle proteolysis. Nat Cell Biol. 2022;24(5):793‐804. 35469018 10.1038/s41556-022-00893-0 PMC9107513

[212] Ferrucci L , Fabbri E . Inflammageing: chronic inflammation in ageing, cardiovascular disease, and frailty. Nat Rev Cardiol. 2018;15(9):505‐522. 30065258 10.1038/s41569-018-0064-2 PMC6146930

[213] Speer T , Dimmeler S , Schunk SJ , Fliser D , Ridker PM . Targeting innate immunity‐driven inflammation in CKD and cardiovascular disease. Nat Rev Nephrol. 2022;18(12):762‐778. 36064794 10.1038/s41581-022-00621-9

[214] Rohm TV , Meier DT , Olefsky JM , Donath MY . Inflammation in obesity, diabetes, and related disorders. Immunity. 2022;55(1):31‐55. 35021057 10.1016/j.immuni.2021.12.013 PMC8773457

[215] Zhang S , Meng Y , Zhou L , et al. Targeting epigenetic regulators for inflammation: mechanisms and intervention therapy. MedComm. 2022;3(4):e173. 36176733 10.1002/mco2.173 PMC9477794

[216] Li Y , Liu H , Xu Q‐S , Du Y‐G , Xu J . Chitosan oligosaccharides block LPS‐induced O‐GlcNAcylation of NF‐κB and endothelial inflammatory response. Carbohydr Polym. 2014;99:568‐578. 24274545 10.1016/j.carbpol.2013.08.082 PMC3843148

[217] Dong X , Shu L , Zhang J , et al. Ogt‐mediated O‐GlcNAcylation inhibits astrocytes activation through modulating NF‐κB signaling pathway. J Neuroinflammation. 2023;20(1):146. 37349834 10.1186/s12974-023-02824-8 PMC10286367

[218] Li X , Gong W , Wang H , et al. O‐GlcNAc transferase suppresses inflammation and necroptosis by targeting receptor‐interacting serine/threonine‐protein kinase 3. Immunity. 2019;50(3):576‐590. 30770249 10.1016/j.immuni.2019.01.007 PMC6426684

[219] He X , Gao J , Peng L , et al. Bacterial O‐GlcNAcase genes abundance decreases in ulcerative colitis patients and its administration ameliorates colitis in mice. Gut. 2021;70(10):1872‐1883. 33310751 10.1136/gutjnl-2020-322468 PMC8458092

[220] Bentsen MA , Mirzadeh Z , Schwartz MW . Revisiting how the brain senses glucose‐and why. Cell Metab. 2019;29(1):11‐17. 30527741 10.1016/j.cmet.2018.11.001 PMC6326855

[221] Cacciatore M , Grasso EA , Tripodi R , Chiarelli F . Impact of glucose metabolism on the developing brain. Front Endocrinol. 2022;13:1047545. 10.3389/fendo.2022.1047545 PMC9816389 36619556

[222] Pinho TS , Verde DM , Correia SC , Cardoso SM , Moreira PI . O‐GlcNAcylation and neuronal energy status: implications for Alzheimer's disease. Ageing Res Rev. 2018;46:32‐41. 29787816 10.1016/j.arr.2018.05.003

[223] Park J , Ha H‐J , Chung ES , et al. O‐GlcNAcylation ameliorates the pathological manifestations of Alzheimer's disease by inhibiting necroptosis. Sci Adv. 2021;7(3):eabd3207. 33523877 10.1126/sciadv.abd3207 PMC7806231

[224] Ephrame SJ , Cork GK , Marshall V , et al. O‐GlcNAcylation regulates extracellular signal‐regulated kinase (ERK) activation in Alzheimer's disease. Front Aging Neurosci. 2023;15:1155630. 37469955 10.3389/fnagi.2023.1155630 PMC10352608

[225] Cook BE , Nag S , Arakawa R , et al. Development of a PET tracer for OGA with improved kinetics in the living brain. J Nucl Med. 2023;64(10):1588‐1593. 37934021 10.2967/jnumed.122.265225

[226] Lee BE , Kim HY , Kim H‐J , et al. O‐GlcNAcylation regulates dopamine neuron function, survival and degeneration in Parkinson disease. Brain. 2020;143(12):3699‐3716. 33300544 10.1093/brain/awaa320 PMC7805798

[227] Hart GW , Huang C‐W . Increased O‐GlcNAcylation prevents degeneration of dopamine neurons. Brain. 2020;143(12):3515‐3518. 33439984 10.1093/brain/awaa398 PMC7805785

[228] Marotta NP , Lin YH , Lewis YE , et al. O‐GlcNAc modification blocks the aggregation and toxicity of the protein α‐synuclein associated with Parkinson's disease. Nat Chem. 2015;7(11):913‐920. 26492012 10.1038/nchem.2361 PMC4618406

[229] Levine PM , Galesic A , Balana AT , et al. α‐Synuclein O‐GlcNAcylation alters aggregation and toxicity, revealing certain residues as potential inhibitors of Parkinson's disease. Proc Natl Acad Sci USA. 2019;116(5):1511‐1519. 30651314 10.1073/pnas.1808845116 PMC6358670

[230] Kolm‐Litty V , Sauer U , Nerlich A , Lehmann R , Schleicher ED . High glucose‐induced transforming growth factor beta1 production is mediated by the hexosamine pathway in porcine glomerular mesangial cells. J Clin Invest. 1998;101(1):160‐169. 9421478 10.1172/JCI119875 PMC508552

[231] Zhang W , Liu J , Tian L , Liu Q , Fu Y , Garvey WT . TRIB3 mediates glucose‐induced insulin resistance via a mechanism that requires the hexosamine biosynthetic pathway. Diabetes. 2013;62(12):4192‐4200. 23990361 10.2337/db13-0312 PMC3837074

[232] Riegger J , Baumert J , Zaucke F , Brenner RE . The hexosamine biosynthetic pathway as a therapeutic target after cartilage trauma: modification of chondrocyte survival and metabolism by glucosamine derivatives and PUGNAc in an ex vivo model. Int J Mol Sci. 2021;22(14):7247. 34298867 10.3390/ijms22147247 PMC8305151

[233] Segel GB , Woodlock TJ , Murant FG , Lichtman MA . Photoinhibition of 2‐amino‐2‐carboxybicyclo[2,2,1]heptane transport by O‐diazoacetyl‐L‐serine. An initial step in identifying the L‐system amino acid transporter. J Biol Chem. 1989;264(28):16399‐16402. 2789219

[234] Earhart RH , Koeller JM , Davis HL . Phase I trial of 6‐diazo‐5‐oxo‐L‐norleucine (DON) administered by 5‐day courses. Cancer Treat Rep. 1982;66(5):1215‐1217. 7083223

[235] Akins NS , Nielson TC , Le HV . Inhibition of glycolysis and glutaminolysis: an emerging drug discovery approach to combat cancer. Curr Top Med Chem. 2018;18(6):494‐504. 29788892 10.2174/1568026618666180523111351 PMC6110043

[236] Xiang J , Chen C , Liu R , et al. Gluconeogenic enzyme PCK1 deficiency promotes CHK2 O‐GlcNAcylation and hepatocellular carcinoma growth upon glucose deprivation. J Clin Invest. 2021;131(8):e144703. 33690219 10.1172/JCI144703 PMC8262473

[237] Liu G , Feng L , Liu X , Gao P , Wang F . O‐GlcNAcylation inhibition upregulates connexin43 expression in the endothelium to protect the tight junction barrier in diabetic retinopathy. Invest Ophthalmol Vis Sci. 2023;64(14):30. 10.1167/iovs.64.14.30 PMC10668625 37982762

[238] Kapuria V , Röhrig UF , Waridel P , et al. The conserved threonine‐rich region of the HCF‐1PRO repeat activates promiscuous OGT:uDP‐GlcNAc glycosylation and proteolysis activities. J Biol Chem. 2018;293(46):17754‐17768. 30224358 10.1074/jbc.RA118.004185 PMC6240873

[239] Gloster TM , Zandberg WF , Heinonen JE , Shen DL , Deng L , Vocadlo DJ . Hijacking a biosynthetic pathway yields a glycosyltransferase inhibitor within cells. Nat Chem Biol. 2011;7(3):174‐181. 21258330 10.1038/nchembio.520 PMC3202988

[240] Andres LM , Blong IW , Evans AC , et al. Chemical modulation of protein O‐GlcNAcylation via OGT inhibition promotes human neural cell differentiation. ACS Chem Biol. 2017;12(8):2030‐2039. 28541657 10.1021/acschembio.7b00232 PMC5850955

[241] Plouin PF . Interactions between antihypertensive agents, serum lipids and cigarette smoking in high risk hypertensive patients. J Hum Hypertens. 1989;3(2):49‐53. 2575176

[242] Lee DE , Lee GY , Lee HM , Choi SY , Lee SJ , Kwon O‐S . Synergistic apoptosis by combination of metformin and an O‐GlcNAcylation inhibitor in colon cancer cells. Cancer Cell Int. 2023;23(1):108. 37268905 10.1186/s12935-023-02954-2 PMC10239094

[243] Taira TM , Ramos‐Junior ES , Melo PH , et al. HBP/O‐GlcNAcylation metabolic axis regulates bone resorption outcome. J Dent Res. 2023;102(4):440‐449. 36749069 10.1177/00220345221141043

[244] Xuefei Y , Dongyan L , Tianming L , Hejuan Z , Jianhua F . O‐linked N‐acetylglucosamine affects mitochondrial homeostasis by regulating Parkin‐dependent mitophagy in hyperoxia‐injured alveolar type II cells injury. Respir Res. 2023;24(1):16. 36647045 10.1186/s12931-022-02287-0 PMC9841680

[245] Zafir A , Readnower R , Long BW , et al. Protein O‐GlcNAcylation is a novel cytoprotective signal in cardiac stem cells. Stem Cells. 2013;31(4):765‐775. 23335157 10.1002/stem.1325 PMC3606688

[246] Dong L , Shen S , Xu Y , et al. Computational studies on the potency and selectivity of PUGNAc derivatives against GH3, GH20, and GH84 β‐N‐acetyl‐D‐hexosaminidases. Front Chem. 2019;7:235. 31111026 10.3389/fchem.2019.00235 PMC6499197

[247] Troiano JA , Potje SR , Graton ME , et al. Pregnancy decreases O‐GlcNAc‐modified proteins in systemic arteries of normotensive and spontaneously hypertensive rats. Life Sci. 2021;266:118885. 33316265 10.1016/j.lfs.2020.118885

[248] You Z , Peng D , Cao Y , et al. P53 suppresses the progression of hepatocellular carcinoma via miR‐15a by decreasing OGT expression and EZH2 stabilization. J Cell Mol Med. 2021;25(19):9168‐9182. 34510715 10.1111/jcmm.16792 PMC8500955

[249] Saha A , Bello D , Fernández‐Tejada A . Advances in chemical probing of protein O‐GlcNAc glycosylation: structural role and molecular mechanisms. Chem Soc Rev. 2021;50(18):10451‐10485. 34338261 10.1039/d0cs01275k PMC8451060

[250] Moriwaki K , Asahi M . Augmented TME O‐GlcNAcylation promotes tumor proliferation through the inhibition of p38 MAPK. Mol Cancer Res. 2017;15(9):1287‐1298. 28536142 10.1158/1541-7786.MCR-16-0499

[251] Papanicolaou KN , Jung J , Ashok D , et al. Inhibiting O‐GlcNAcylation impacts p38 and Erk1/2 signaling and perturbs cardiomyocyte hypertrophy. J Biol Chem. 2023;299(3):102907. 36642184 10.1016/j.jbc.2023.102907 PMC9988579

[252] Yu F , Yang S , Ni H , et al. O‐GlcNAcylation regulates centrosome behavior and cell polarity to reduce pulmonary fibrosis and maintain the epithelial phenotype. Adv Sci. 2023:e2303545. 10.1002/advs.202303545 PMC10754140 37963851

[253] Yang YR , Song M , Lee H , et al. O‐GlcNAcase is essential for embryonic development and maintenance of genomic stability. Aging Cell. 2012;11(3):439‐448. 22314054 10.1111/j.1474-9726.2012.00801.x

[254] Lee TN , Alborn WE , Knierman MD , Konrad RJ . Alloxan is an inhibitor of O‐GlcNAc‐selective N‐acetyl‐beta‐D‐glucosaminidase. Biochem Biophys Res Commun. 2006;350(4):1038‐1043. 17045574 10.1016/j.bbrc.2006.09.155

[255] Whitworth GE , Macauley MS , Stubbs KA , et al. Analysis of PUGNAc and NAG‐thiazoline as transition state analogues for human O‐GlcNAcase: mechanistic and structural insights into inhibitor selectivity and transition state poise. J Am Chem Soc. 2007;129(3):635‐644. 17227027 10.1021/ja065697o

[256] Alonso J , Schimpl M , van Aalten DMF . O‐GlcNAcase: promiscuous hexosaminidase or key regulator of O‐GlcNAc signaling? J Biol Chem. 2014;289(50):34433‐34439. 25336650 10.1074/jbc.R114.609198 PMC4263850

[257] Dorfmueller HC , Borodkin VS , Schimpl M , Shepherd SM , Shpiro NA , van Aalten DMF . GlcNAcstatin: a picomolar, selective O‐GlcNAcase inhibitor that modulates intracellular O‐glcNAcylation levels. J Am Chem Soc. 2006;128(51):16484‐16485. 17177381 10.1021/ja066743n PMC7116141

[258] Liu T , Xia M , Zhang H , et al. Exploring NAG‐thiazoline and its derivatives as inhibitors of chitinolytic β‐acetylglucosaminidases. FEBS Lett. 2015;589(1):110‐116. 25436416 10.1016/j.febslet.2014.11.032

[259] Kim C , Nam DW , Park SY , et al. O‐linked β‐N‐acetylglucosaminidase inhibitor attenuates β‐amyloid plaque and rescues memory impairment. Neurobiol Aging. 2013;34(1):275‐285. 22503002 10.1016/j.neurobiolaging.2012.03.001

[260] Li X , Han J , Bujaranipalli S , et al. Structure‐based discovery and development of novel O‐GlcNAcase inhibitors for the treatment of Alzheimer's disease. Eur J Med Chem. 2022;238:114444. 35588599 10.1016/j.ejmech.2022.114444

[261] Désiré J , Foucart Q , Poveda A , et al. Synthesis, conformational analysis and glycosidase inhibition of bicyclic nojirimycin C‐glycosides based on an octahydrofuro[3,2‐b]pyridine motif. Carbohydr Res. 2022;511:108491. 34953389 10.1016/j.carres.2021.108491

[262] Yuzwa SA , Macauley MS , Heinonen JE , et al. A potent mechanism‐inspired O‐GlcNAcase inhibitor that blocks phosphorylation of tau in vivo. Nat Chem Biol. 2008;4(8):483‐490. 18587388 10.1038/nchembio.96

[263] Selnick HG , Hess JF , Tang C , et al. Discovery of MK‐8719, a potent O‐GlcNAcase inhibitor as a potential treatment for tauopathies. J Med Chem. 2019;62(22):10062‐10097. 31487175 10.1021/acs.jmedchem.9b01090

[264] Mikesh LM , Ueberheide B , Chi A , et al. The utility of ETD mass spectrometry in proteomic analysis. Biochim Biophys Acta. 2006;1764(12):1811‐1822. 17118725 10.1016/j.bbapap.2006.10.003 PMC1853258

[265] Zhao P , Viner R , Teo CF , Boons G‐J , Horn D , Wells L . Combining high‐energy C‐trap dissociation and electron transfer dissociation for protein O‐GlcNAc modification site assignment. J Proteome Res. 2011;10(9):4088‐4104. 21740066 10.1021/pr2002726 PMC3172619

[266] Ma J , Wu C , Hart GW . Analytical and biochemical perspectives of protein O‐GlcNAcylation. Chem Rev. 2021;121(3):1513‐1581. 33416322 10.1021/acs.chemrev.0c00884

[267] Cameron A , Giacomozzi B , Joyce J , et al. Generation and characterization of a rabbit monoclonal antibody site‐specific for tau O‐GlcNAcylated at serine 400. FEBS Lett. 2013;587(22):3722‐3728. 24113653 10.1016/j.febslet.2013.09.042

[268] Fujiki R , Hashiba W , Sekine H , et al. GlcNAcylation of histone H2B facilitates its monoubiquitination. Nature. 2011;480(7378):557‐560. 22121020 10.1038/nature10656 PMC7289526

[269] Pathak S , Borodkin VS , Albarbarawi O , Campbell DG , Ibrahim A , van Aalten DM . O‐GlcNAcylation of TAB1 modulates TAK1‐mediated cytokine release. EMBO J. 2012;31(6):1394‐1404. 22307082 10.1038/emboj.2012.8 PMC3321193

[270] Shan H , Sun J , Shi M , et al. Generation and characterization of a site‐specific antibody for SIRT1 O‐GlcNAcylated at serine 549. Glycobiology. 2018;28(7):482‐487. 29688431 10.1093/glycob/cwy040

[271] Póvoa P , Coelho L , Dal‐Pizzol F , et al. How to use biomarkers of infection or sepsis at the bedside: guide to clinicians. Intens Care Med. 2023;49(2):142‐153. 10.1007/s00134-022-06956-y PMC9807102 36592205

[272] Phoomak C , Silsirivanit A , Wongkham C , Sripa B , Puapairoj A , Wongkham S . Overexpression of O‐GlcNAc‐transferase associates with aggressiveness of mass‐forming cholangiocarcinoma. Asian Pac J Cancer Prev. 2012;13:101‐105. 23480751

[273] Starska K , Forma E , Brzezińska‐Błaszczyk E , et al. Gene and protein expression of O‐GlcNAc‐cycling enzymes in human laryngeal cancer. Clin Exp Med. 2015;15(4):455‐468. 25315705 10.1007/s10238-014-0318-1 PMC4623075

[274] Zhu Q , Zhou L , Yang Z , et al. O‐GlcNAcylation plays a role in tumor recurrence of hepatocellular carcinoma following liver transplantation. Med Oncol. 2012;29(2):985‐993. 21461968 10.1007/s12032-011-9912-1

